# Genomic Differences Between the Sexes in a Fish Species Seen Through Satellite DNAs

**DOI:** 10.3389/fgene.2021.728670

**Published:** 2021-09-30

**Authors:** Carolina Crepaldi, Emiliano Martí, Évelin Mariani Gonçalves, Dardo Andrea Martí, Patricia Pasquali Parise-Maltempi

**Affiliations:** ^1^ Departamento de Biologia Geral e Aplicada, Instituto de Biociências (IB), Universidade Estadual Paulista (UNESP), Rio Claro, Brazil; ^2^ Laboratorio de Genética Evolutiva, Instituto de Biología Subtropical (IBS), Universidad Nacional de Misiones (UNaM), CONICET, Posadas, Argentina

**Keywords:** satellitome, concerted evolution, satDNA evolution, neotropical fish, fish sex chromosomes, megaleporinus, anostomidae

## Abstract

Neotropical fishes have highly diversified karyotypic and genomic characteristics and present many diverse sex chromosome systems, with various degrees of sex chromosome differentiation. Knowledge on their sex-specific composition and evolution, however, is still limited. Satellite DNAs (satDNAs) are tandemly repeated sequences with pervasive genomic distribution and distinctive evolutionary pathways, and investigating satDNA content might shed light into how genome architecture is organized in fishes and in their sex chromosomes. The present study investigated the satellitome of *Megaleporinus elongatus*, a freshwater fish with a proposed Z_1_Z_1_Z_2_Z_2_/Z_1_W_1_Z_2_W_2_ multiple sex chromosome system that encompasses a highly heterochromatic and differentiated W_1_ chromosome. The species satellitome comprises of 140 different satDNA families, including previously isolated sequences and new families found in this study. This diversity is remarkable considering the relatively low proportion that satDNAs generally account for the *M. elongatus* genome (around only 5%). Differences between the sexes in regards of satDNA content were also evidenced, as these sequences are 14% more abundant in the female genome. The occurrence of sex-biased signatures of satDNA evolution in the species is tightly linked to satellite enrichment associated with W_1_ in females. Although both sexes share practically all satDNAs, the overall massive amplification of only a few of them accompanied the W_1_ differentiation. We also investigated the expansion and diversification of the two most abundant satDNAs of *M. elongatus,* MelSat01-36 and MelSat02-26, both highly amplified sequences in W_1_ and, in MelSat02-26’s case, also harbored by Z_2_ and W_2_ chromosomes. We compared their occurrences in *M. elongatus* and the sister species *M. macrocephalus* (with a standard ZW sex chromosome system) and concluded that both satDNAs have led to the formation of highly amplified arrays in both species; however, they formed species-specific organization on female-restricted sex chromosomes. Our results show how satDNA composition is highly diversified in *M. elongatus*, in which their accumulation is significantly contributing to W_1_ differentiation and not satDNA diversity per se. Also, the evolutionary behavior of these repeats may be associated with genome plasticity and satDNA variability between the sexes and between closely related species, influencing how seemingly homeologous heteromorphic sex chromosomes undergo independent satDNA evolution.

## Introduction

Among the repetitive fraction of a eukaryotic genome, satellite DNAs (satDNAs) are one of the most abundant elements, characterized as tandemly organized sequences that can be amplified into multiple copies in the genome (Charlesworth et al., 1994; Plohl et al., 2012). Selective forces act loosely on satDNAs, as they are prone to accumulate random mutations and rapidly diverge from each other. This ultimately leads to large arrays of diversified satellite composition, as they can vary in sequence length, nucleotide composition, genomic position and chromosome distribution (Ugarković and Plohl, 2002; Garrido-Ramos, 2017). Repeats within the same satDNA family, however, show lower divergence rates as they do not evolve independently, but via what is called “concerted evolution” (Dover, 1982; Elder and Turner, 1995; Thakur et al., 2021). These satDNAs are submitted to the process of molecular drive, which causes sequence homogenization for species-specific mutations and results in repetitions evolving in concert with each other (Dover, 1982; Dover, 1986; Elder and Turner, 1995; Ugarković and Plohl, 2002; Thakur et al., 2021). The rates at which these sequences expanded, homogenized and eventually fixed in the genome vary for each satDNA family. These levels of variation depend on a number of factors, such as mutation rate, array size and structure, chromosomal structure and recombination rates (Ohta and Dover, 1984; Elder and Turner, 1995; Plohl et al., 2012). SatDNA evolution encompasses the duality of combining stable homogeneous arrays fixed in a genome and the high dynamism of rapidly replaceable sequences (i.e., turnover) (Ugarković and Plohl, 2002). These features still place satDNA evolutionary perspective under necessary evaluation. Nonetheless, what is certainly known so far is how the evolutionary mechanisms governing these sequences are different in comparison to other genomic elements (Plohl et al., 2012; Thakur et al., 2021).

Heterochromatin is a poorly understood genomic component, perhaps given the specific nature of its repetitive content (composed mostly of satDNAs) (Charlesworth et al., 1994; Garrido-Ramos, 2017). Sex chromosomes are a good example of genomic entities that can experience heterochromatin expansions and contractions along their evolution, as they can be submitted to rapid diversification after colonization by repetitive sequences and they might gradually differentiate from their homologs, becoming heteromorphic sex chromosomes (Chalopin et al., 2015; Palacios-Gimenez et al., 2015; Wright et al., 2016; Yano et al., 2016; Sember et al., 2018; Charlesworth, 2021; Kratochvíl et al., 2021). The rapid spread of repetitive sequences in a heterogametic (Y or W) sex chromosome may occur during the initial phase of its existence and it may facilitate the expansion of regions with ceased recombination, which further helps XY or ZW counterparts to differentiate from each other (Charlesworth, 1991; Bergero and Charlesworth, 2009; Bachtrog et al., 2011; Schartl et al., 2016).

Teleost fishes compose the most speciose group of vertebrates, which present equally diversified spectrum of sex chromosome occurrences scattered throughout the taxa (Devlin and Nagahama, 2002; Volff, 2005; Godwin and Roberts, 2018; Sember et al., 2021). Teleosts display variable degrees of molecular differentiation in their sex chromosomes, even among closely related species; which contrasts with the more uniform sex systems found in birds and therian mammals (Ellegren, 2011; Schartl et al., 2016; Charlesworth, 2019). Having said that, not much is known about what molecular mechanisms underlie this large compendium of sex chromosome occurrences or the evolutionary dynamics and genomic features of teleosts as a whole.

*Megaleporinus* is a Neotropical fish genus with a conserved karyotype of 2n = 54 chromosomes, female heterogamety (ZW) and, in *M. elongatus* case, a hypothetical Z_1_Z_1_Z_2_Z_2_/Z_1_W_1_Z_2_W_2_ multiple sex chromosome system (Parise-Maltempi et al., 2007; Parise-Maltempi et al., 2013). The heteromorphic sex chromosomes for both systems (W and W_1_) are highly heterochromatic and they constitute the largest elements in the female karyotype, which makes the understanding of *Megaleporinus* species genomic traits and sex chromosome systems a coveted approach (Nakayama et al., 1994; Parise-Maltempi et al., 2007; Hashimoto et al., 2009; Ramirez et al., 2017a; Ramirez et al., 2017b; Dulz et al., 2020). Heterochromatin content of the genus has sparked interest in previous works that specifically targeted repetitive DNA occurrence in the heteromorphic sex chromosomes (da Silva et al., 2012; Marreta et al., 2012; de Borba et al., 2013; Parise-Maltempi et al., 2013; Poltronieri et al., 2014). However, only recently was it possible to effectively quantify satDNA content and trace their evolutionary features within *Megaleporinus* (Utsunomia et al., 2019; Crepaldi and Parise-Maltempi, 2020).

Thus, in the present study we used low-coverage genomic DNA data yielded from Illumina paired-end sequencing to assess and further investigate the genomic organization of the highly diversified satellitome in *Megaleporinus elongatus*, firstly presented by Crepaldi and Parise-Maltempi (2020). Cytogenomic and haplotype analyses were also used to further investigate possible sex-specific patterns of these satDNAs and their evolutionary pathways. We complemented our survey with an in-depth analysis of W_1_-located satDNAs in both *M. elongatus* and its sister species *M. macrocephalus*, focusing specifically on the two most abundant and quantitatively relevant elements of *M. elongatus* satellitome. By complementing previous satDNA and FISH results (Crepaldi and Parise-Maltempi, 2020) with new additions to satellitome data and haplotype networks, we managed to trace a possible evolutionary pathway for these satDNAs in *M. elongatus* and understand how they contributed to the recent differentiation of the heteromorphic sex chromosomes and their possible role in the multiple sex chromosome differentiation. Altogether, our study aimed to integrate thorough genomic analyses to previously published data for the *Megaleporinus* genus and to provide new information regarding the evolution of repetitive genomic composition in the group, as well as satDNA evolutionary differences between the sexes and closely related species.

## Materials and Methods

### Species Sampling and Chromosome Preparation

Chromosomal preparations and tissue samples from three males and three females from *Megaleporinus elongatus* (Anostomidae) were analyzed*.* All samples were already available at the Animal Cytogenetics Laboratory in UNESP Rio Claro, Brazil from previous studies (da Silva et al., 2012; Marreta et al., 2012; Parise-Maltempi et al., 2013; Crepaldi and Parise-Maltempi, 2020). All procedures for sampling, material handling and analysis were authorized and approved by the Animal Ethics Committee (Comitê de Ética no Uso de Animais (CEUA) (protocol number 3524, approval code 09/2017), by the Brazilian Institute of Environment and Renewable Natural Resources (Instituto Brasileiro do Meio Ambiente e Recursos Naturais Renováveis - IBAMA) (19833-1) and the Brazilian College of Animal Experimentation (Colégio Brasileiro de Experimentação Animal - 016/04-CEEA). Mitotic chromosomes were obtained from anterior kidney cell suspensions, according to Foresti and Toledo (1981).

### Genome Sequencing and Computational Satellitome Analysis

In the present study, previously sequenced libraries from each sex of *M. elongatus* using Illumina^®^ Hiseq™ 2000 platform (female) and Illumina^®^ Hiseq™ 4000 platform (male) (Crepaldi and Parise-Maltempi, 2020) were used, which provided 1.9 Gb of sequence data and yielded 19,289,312 paired-end trimmed reads for the female, and 1.8 Gb of data and 17,837,098 paired-end trimmed reads for the male library.

The sequenced libraries were used for comparative analysis regarding their satDNA content. We focused both on the species’ satellitome as a whole and on the differences between the sexes, in search for sequences that could be more representative in the female and probably enriched in the heteromorphic W_1_ sex chromosome. To perform a high-throughput analysis, the satMiner bioinformatic protocol for satDNA prospection in both libraries was used (Ruiz-Ruano et al., 2016) available at GitHub (https://github.com/fjruizruano/satminer). The satMiner protocol uses several rounds of clustering in RepeatExplorer (Novák et al., 2013) to identify and extract satDNA sequences, and each round includes filtering out reads matching previously assembled contigs with deconseq 0.4.3 (Schmieder and Edwards, 2011), in order to identify and extract as many sequences as possible, even with low abundance in the genome. We then started with a library sampling of 200,000 reads, incrementing this number by two in each consequent round of RepeatExplorer clustering. For each round, we selected clusters with spherical shaped graphs for putative satDNA. Each selected cluster was manually analyzed for their internal contig structure and tandem repetitions were investigated using the dotplot tool implemented in Geneious v4.8 (Drummond et al., 2015) and Tandem Repeat Finder (TRF) (Benson, 1999).

The clustering and filtering steps were repeated 13 times for the female library and 9 times for the male one, adding new filtered reads in each iteration until we could no longer detect new satDNAs in neither. A parallel homology search was performed in both male and female rounds using previously detected satDNA consensus sequences by Crepaldi and Parise-Maltempi (2020) as a custom library to match ones that might have previously been isolated and physically mapped.

After satDNA mining, all-against-all alignments with RepeatMasker (Smit et al., 2013) were performed to search for homologous satDNAs, and by comparing all monomers from all clusters we were able to classify them into superfamilies, families or variants (Ruiz-Ruano et al., 2016). Non-redundant consensus library was used for each satDNA family to check for possible similarities with published sequences deposited in Genbank and Repbase employing BLAST (http://www.ncbi.nlm.gov/Blast/) and Censor (http://www.girinst.org/censor) searches.

All satDNA families were numbered in order of decreasing abundance in the female genome and assigned following the nomenclature proposed by Ruiz-Ruano et al. (2016). All satDNAs that were previously isolated by Crepaldi and Parise-Maltempi (2020) were properly renamed also following this criterion. Sequences are deposited in GenBank under accession numbers MZ546645–MZ546784.

RepeatMasker with rmblast engine was used to determine abundance and average nucleotide divergence (Kimura-2-parameter, K2P) for each variant in both sexes. We estimated the genomic abundance for every satDNA in the male and female libraries as the number of nucleotides aligned to the reference consensus divided by the library size (in bp). With this data we generated repeat landscapes for the relative abundance (Y-axis) at 1% intervals of K2P distance from the consensus (X-axis), using the script calcDivergencFromAlign.pl (from RepeatMasker utils). A subtractive landscape was subsequently generated to evaluate which satDNA families differ between both libraries, in turn providing the first indications of which satDNA are more prominent in one sex in comparison to the other.

### Physical Mapping via FISH in Male Metaphases

We selected the same previously isolated and amplified 52 satDNAs in the female (Crepaldi and Parise-Maltempi, 2020) and amplified them via PCR in *M. elongatus* males following the protocol described by the authors. The PCR products were confirmed via Sanger sequencing.

The sequences of each satDNA obtained through PCR were labeled by nick translation with digoxigenin-11-dUTP (Roche^®^) or biotin-14-dATP (Invitrogen^®^). Fluorescence *in situ* hybridization (FISH) experiments were performed following the method described by Pinkel et al. (1986), with small adjustments described by Crepaldi and Parise-Maltempi (2020). The resulting slides were visualized under an Olympus^®^ BX51 fluorescence microscope, with a digital camera Olympus^®^ DP71 attached, and the images were captured using the DP Controller camera software. For each slide, a minimum of 20 metaphases were analyzed and photographed to confirm the FISH results.

### Sex-Biased Ratio

The different enrichment of all satDNAs across the sexes was determined by generating a female to male ratio as we calculated the quotient between the abundance values of each satDNA family. This data complemented the subtractive landscape by providing more between-sexes differences, as satDNA families with Female/Male (F/M) ratio higher than one were considered more abundant in females (as the threshold to determine it as more prevalent in this sex).

RepeatProfiler pipeline (Negm et al., 2021) was applied to generate comparative variant-enhanced profiles of selected satDNAs, which provide a summary of variant sites that are relative to the consensus sequences and may uncover sex-specific signatures of sequence variants and possible point mutations in satDNAs of interest. The 22 satDNA families that are most enriched in each sex considering the F/M ratio were selected for profiling. RepeatProfiler relies on Bowtie2 (Langmead and Salzberg, 2012) for mapping the reads to the consensus monomers of the selected sequences and the pipeline generated a PDF file for each selected satellite with the variant-enhanced profiles for both sexes as an output. We applied Bowtie2 default parameters for RepeatProfiler.

After analyzing the resulting profiles, we generated individual landscapes for each selected female-biased satDNAs to confirm different amplification and divergence of their copies in male and female genomes.

### Retrieving satDNA Monomers From Raw Reads of *M. elongatus* and *M. macrocephalus*

Comparative cytogenetic analysis of the two most abundant satDNAs (MelSat01-36 and MelSat02-26) revealed particular characteristics that prompted us to investigate them further, such as differences in clustered patterns in the W chromosome in *M. macrocephalus* and W_1_ in *M. elongatus* and occurrence in autosomal pairs in both males and females. MelSat02-26 is specifically interesting given it is a fragment of Le*Spe*I, a repetitive marker used for the *M. elongatus* hypothetical multiple sex chromosome system (Parise-Maltempi et al., 2007; Crepaldi and Parise-Maltempi, 2020).

Monomers from Illumina reads representing MelSat01-36 and MelSat02-26 were extracted from both sexes of *M. elongatus* and *M. macrocephalus* libraries. Thus, Minimum Spanning Trees (MSTs) of these satDNA families were generated to trace the diversification patterns of copies between sexes and species. First, we mapped reads from raw Illumina libraries from the two selected satDNA, of both sexes of *M. elongatus* and *M. macrocephalus*, with a custom script (https://github.com/fjruizruano/satminer/blob/master/mapping_blat_gs.py).

Libraries of *M. macrocephalus* were retrieved from Sequence Read Archive (SRA) under the accession numbers SRR7263033 and SRR7263034. Mapped reads were then extracted from SAM files and aligned separately using MUSCLE (Edgar, 2004) with default parameters. The resulting alignment files of each satDNA were used as input in PHYLOViZ 2.0 (Nascimento et al., 2017) to construct MSTs on the bases of pairwise differences. RepeatMasker was used to determine abundance and average nucleotide divergence (K2P) for MelSat01-36 and MelSat02-26 in both sexes of *M. macrocephalus*. Then, the genomic abundance of these two satDNAs in reference to this species library sizes (in bp) was calculated, and this data was used to generate comparative landscapes between *M. elongatus* and *M. macrocephalus.*

## Results

### General Satellitome Analysis in *M. elongatus*

A total of 140 different satDNA families (308 variants) were uncovered for *M. elongatus* as a whole, with a predominance of short repeat unit lengths (RUL) ranging from 11 to 245 bp (average of 43 bp) for both sexes. A + T content of consensus sequences varied between 30.8 and 80.4% (60% on average), which indicated a slight tendency towards A + T rich content for the majority of satDNAs (Supplementary Table S1). The homology analysis of repeat units revealed only one satDNA superfamily (SF1) comprising MelSat09-60 and MelSat113-60 (76.7%) (Supplementary Figure S1) and present in both sexes with relatively similar abundances. Both sequences share the same RUL (60 bp) and have high divergence values in both sexes (Supplementary Table S1).

SatDNA genome proportion ranged from 0.0001 to 0.484%, with the three most abundant satDNAs (MelSat01-36, MelSat02-26 and MelSat03-177) showing an abundance higher than 0.3%. SatDNA abundance in the present work regards the values determined by RepeatMasker that applied a substantial amount of reads in comparison to the pool of randomly selected reads analysed previously (and solely via RepeatExplorer output) by Crepaldi and Parise-Maltempi (2020). MelSat01-36 and MelSat02-26 remained the two most abundant families as previously described (Crepaldi and Parise-Maltempi, 2020) (0.484 and 0.413% respectively), but MelSat03-177 (previously the family MelSat07-177 located in the centromeric area) ranked third in abundance after this thorough analysis, representing 0.338% of the genome.

Therefore, the satellitome of *M. elongatus* consists primarily of the top three most prevalent satDNA, which that comprise almost 25% of the whole compendium of satellites, in addition to 10 families with abundance between 0.1 and 0.3% and a remainder of rare or very low abundant sequences (Supplementary Table S1). Overall divergence values were relatively variable for the species as a whole, ranging from 2.85 to 33.02% (average divergence for the species was 10.73%).

Searches in GenBank resulted in 46 families from *M. elongatus* with positive results with deposited *M. macrocephalus* satDNA sequences (Utsunomia et al., 2019). BLAST results and subsequent alignments for MelSat03-177 showed high similarities with centromeric sequences from other Characiformes (Utsunomia et al., 2017, Utsunomia et al.2019). MelSat40-52 (previously named MelSat49-52 (Crepaldi and Parise-Maltempi, 2020) also showed positive results for other satellite sequences in Characiformes, a conserved sequence with active transcription in *Characidium gomesi* (dos Santos et al., 2021).

FISH analysis was performed in male metaphases for *M. elongatus* for the same satDNA previously mapped in the female (Crepaldi and Parise-Maltempi, 2020). From the 52 satDNAs positively amplified via PCR, only 5 (MelSart02-26, MelSat22-34, MelSat29-121, MelSat44-52 and MelSat52-38) successfully hybridized in this sex (Supplementary Figure S2), in one autosomal pair each and, for MelSat02-26, in Z_2_Z_2_. Each satDNA presented a single band in the chromosomes, and positioned either in the telomeric or in the centromeric regions. No satDNA was mapped exclusively in the male, as all five presented FISH bands in female chromosomes (Crepaldi and Parise-Maltempi, 2020).

### Satellitome Differences Between the Sexes

The total satDNA composition corresponded to 4.83 and 4.23% of the female and male genomes, respectively. Average satDNA family divergence was lower in the female (10.38%) than in the male genome (11.07%), and some satDNAs had quite higher divergence values for the male in comparison to the female, such as MelSat66-46 (33.02 and 7.38%, for male and female respectively) and MelSat64-64 (22.44 and 6.34%). It was later found these satDNAs are highly female-biased according to the F/M ratio (Supplementary Table S1) and with insignificant abundance values for the male library, which sparked our interest to investigate further these differences, described hereinafter.

**FIGURE 1:**
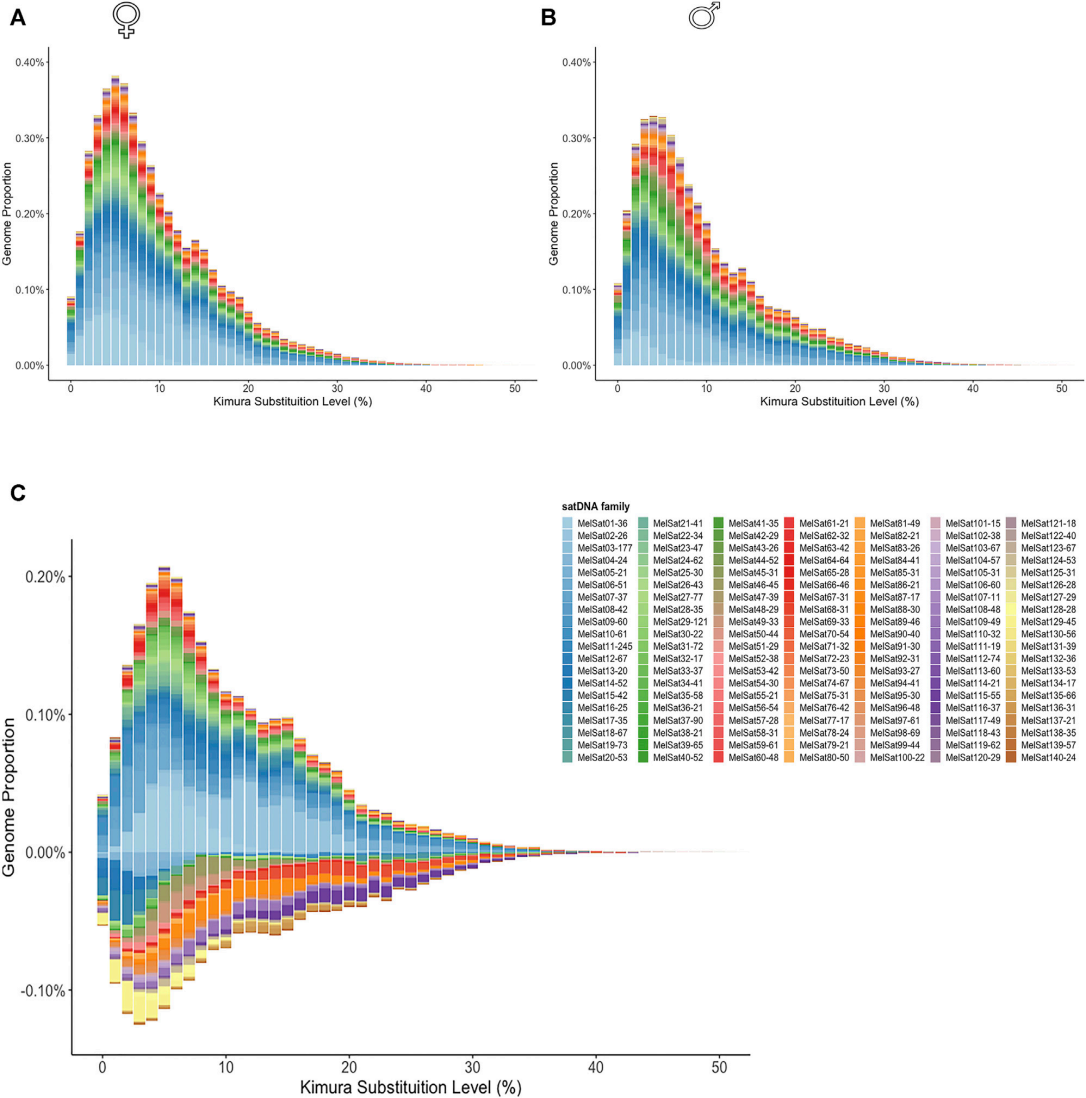
Repeat landscapes (abundance vs. divergence) for satDNAs identified in female **(A)** and male **(B)** *M. elongatus*. The graphs show, for each color-coded element, the sequence divergence according to Kimura distance (x-axis) in relation to their copies in the genome (y-axis). Copies clustered to the left (lower divergence) potentially correspond to recent copies occurring in the genome. A subtractive landscape **(C)** was obtained by subtracting male library (negative values) from the female one (positive values). Notice the diversity of satDNA families also present in the male genome in the subtractive landscape.

We generated individual repeat landscapes for female and male *M. elongatus* (Figure 1A and Figure 1B, respectively) and a subtractive landscape (Figure 1C). The subtractive landscape revealed higher proportions of several abundant satDNA families in the female library, such as MelSat01-36 and MelSat02-26, which are located in the W1 sex chromosome (Crepaldi and Parise-Maltempi, 2020). Interestingly, we could also confirm that the male library presents a higher variety of male-biased satDNAs in comparison to the female (Figure 1C), which are located in the W_1_ sex chromosome (Crepaldi and Parise-Maltempi, 2020). Interestingly, also through the subtractive repeat landscape we could confirm that the male library presents a higher variety of male-biased satDNAs in comparison to the female (Figure 1C).

The computational analysis revealed that, except for MelSat131-39, which is present in the female genome only, all remaining 139 satDNA families are shared between the sexes. From these, however, 124 are differently enriched across sexes as 45 satDNAs had a F/M ratio higher than 1, suggesting an enrichment in the female library, while 79 were deemed male-biased (F/M ratio lower than 1). Despite having less overall female-biased satDNAs in numbers, several families had ratios ranging from 13 up to 347 in the female, denoting an expressive enrichment in this sex compared to the male library (Supplementary Table S2). In short, the female library has the most abundant satDNAs, enriched in the W_1_ (Crepaldi and Parise-Maltempi, 2020); however, the greater diversity of satDNAs is male-biased, comprised mostly of the very rare and less abundant families of the satellitome.

### Variant Profiles for Sex-Biased satDNAs

RepeatProfiler pipeline was applied in order to evaluate the sequences of the most sex-biased satDNAs and how they might differ between the sexes. The output provided variant-enhanced profiles for each selected satDNA, summarizing sex-specific signatures for some sequences (Supplementary Figures S3, S4). All male-biased satDNAs (Supplementary Figure S3) have relatively similar divergent values for both sexes and, with few exceptions (such as MelSat140-24, MelSat137-21 and MelSat92-31), they did not present such apparent differences between sexes as the female-biased ones, showing some conservatism in comparison with male and female profiles. Female-biased satDNAs (Supplementary Figure S4), on the other hand, showed some reoccurring patterns: 1) most have discrepant divergent values between the sexes (with high divergent values for the male) and are either absent in males or have incomplete profiles for this sex, since the aligner failed to map high-divergent reads to consensus; 2) comparable satDNAs were prone to variation in male variants, with particular positions exhibiting almost fixed copies, since mutation profiles differed between the sexes when abundance values dropped; and 3) the only two satDNAs that presented relative homogeneity between the sexes are MelSat01-36 and MelSat02-26 (the two most abundant satDNAs in the genome).

We complemented the variant profiles results by generating individual repeat landscapes (Supplementary Figure S5) for the top most female-biased satDNAs, to check for differential amplification and divergence values between the sequences especially on satDNAs that are most likely specific to the female. The landscapes also evidenced the higher abundance in the female and contrasting divergences for these sequences in the male genome. Some satDNAs, such as MelSat57-28, MelSat112-74 and MelSat123-67 showed monomers with low divergences in the female and shared higher divergences between the sexes, indicating highly divergent copies comparing the genomes.

### Tracing the Amplification of W-Localized satDNAs in *M. elongatus* and *M. macrocephalus*

**FIGURE 2:**
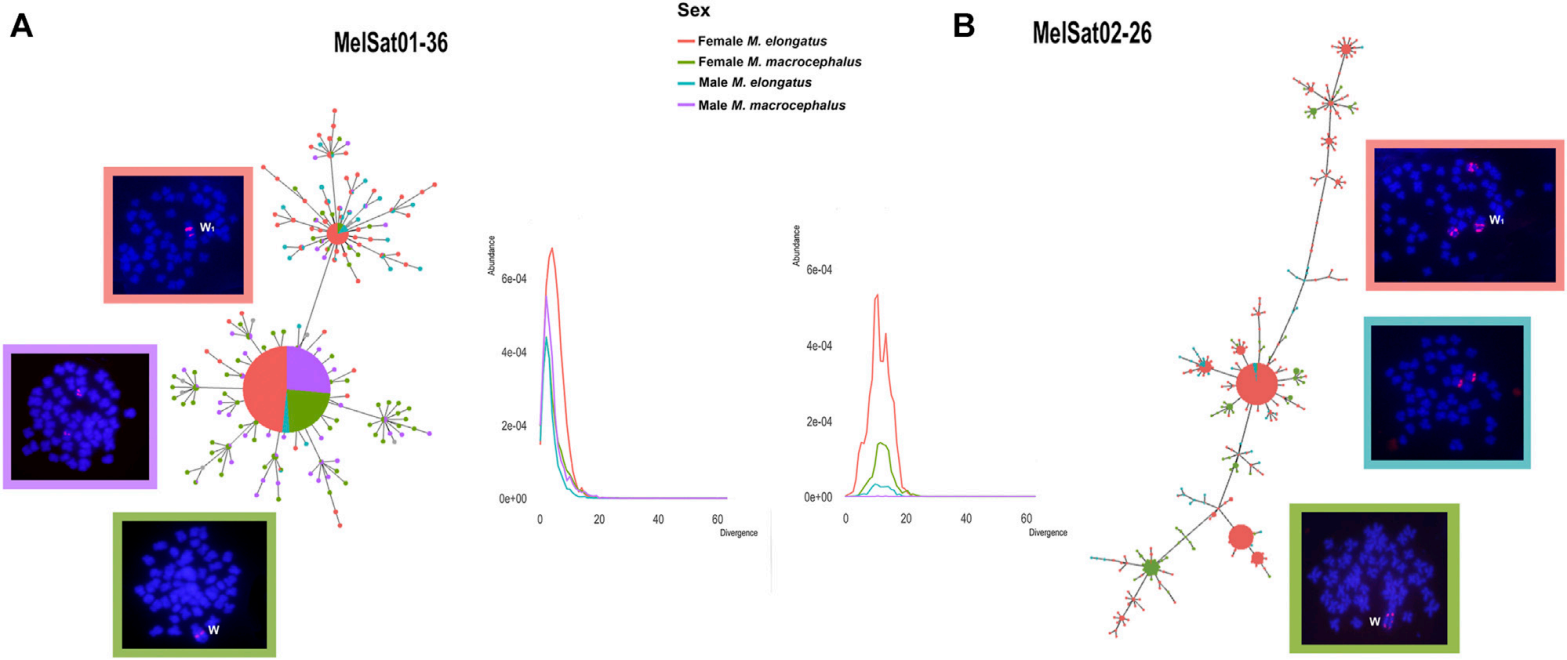
MSTs showing the relationships between isolated monomers for MelSat01-36 **(A)** and MelSat02-26 **(B)** retrieved from *M. elongatus* and *M. macrocephalus* males and females. Respective FISH results and the heteromorphic sex chromosomes W and W_1_ are indicated. Male FISH results for *M. elongatus* and *M. macrocephalus* are absent for MelSat01-36 and MelSat02-26 as no FISH signals were obtained, respectively. Abundance x divergence landscapes that shows the respective similar origins in both species are also indicated, but the substantial abundance in *M. elongatus* female for both satDNAs is apparent. Note the different divergences between the satDNAs, as MelSat02-26 has larger divergence values than MelSat01-36 and a rather dispersed organization in comparison to the latter.

MSTs for MelSat01-36 and MelSat02-26 were generated and complemented with comparative landscapes for these sequences individually in order to integrate their abundance and divergence values to the analysis. Different MSTs and line plots were obtained for each satDNA (Figure 2).

The topology for MelSat01-36 confirms the relatedness and conservatism of this satDNA, especially when considering the larger haplotype shared between both species and its co-localized pattern in FISH results (Figure 2A). Furthermore, due to its low divergence values in both species, it most likely had a common origin in both genomes and an equally recent amplification in the heteromorphic sex chromosomes in the females.

Some discrepancies, however, are notable for *M. elongatus*. Firstly, clusterisation is absent in the male (no FISH signals). In addition to the fact that it has the least abundance in the male of *M. elongatus* of all four analyzed individuals, it is probably gradually losing copies in its genome. This coincides with another *M. elongatus* conspicuous characteristic, perhaps the most noticeable: the female copies. MelSat01-36 is not only more abundantly represented in the female genome, but it is also presented in two separate clusters in the heteromorphic chromosome (Figure 2A).

For MelSat02-26, the resulting MSTs displayed a more divergent pattern and a different evolutionary scenario for this satDNA (Figure 2B) was observed. Firstly, no monomers were retrieved from the male *M. macrocephalus*, as its abundance was virtually non-existent in this individual. For the remaining copies, all three (female *M. macrocephalus,* male and female *M. elongatus*) presented similar divergence with rather higher values, which implies that MelSat02-26 comprised an older satDNA especially in comparison to MelSat01-36.

Both sexes of *M. elongatus* share autosomal clusters and its origins appear concomitant in male and female, especially considering it is clustered in the same autosomal pair and putative multiple sex chromosomes in both sexes (Parise-Maltempi et al., 2007; Crepaldi and Parise-Maltempi, 2020). There is a distinct spike in abundance for *M. elongatus* female in the line plot for this satDNA (Figure 2B), which, paired with the clustered pattern in FISH, shows the substantial amplification in the heteromorphic sex chromosome, which also occurred long since. It presents a different amplification trajectory between the species: although *M. macrocephalus* female also presents clusterisation in its W, it is not as considerable as in *M. elongatus* W_1_ and it does not share any copies with *M. macrocephalus* male.

## Discussion

The present study provides relevant information regarding the satDNA content of *Megaleporinus elongatus* and its genomic features, adding notable details to the first glimpse of these sequences previously published by our group (Crepaldi and Parise-Maltempi, 2020). Using more thorough methods for satDNA prospection, we expanded the array of satellite features in the species, compared satellitomes between the sexes and uncovered the evolutionary pathway of the most abundant (and most quantitively relevant) satDNAs in the genome and their differences between species.

Firstly, some distinct characteristics are responsible for the *M. elongatus* satellitome as a whole, which showed small and highly diversified sequences, mostly recently amplified (low divergence) and with general low abundance. These characteristics were also found in other Characiformes, such as *Astyanax* (Silva et al., 2017), *Megaleporinus macrocephalus* (Utsunomia et al., 2019) and *Characidium gomesi* (Serrano-Freitas et al., 2020).

The disparity of a large satDNA diversity in *M. elongatus* (140 families) and such a scarce occurrence result in very rare sequences, organized in small arrays and with a dispersed organization in the genome. The massively amplified MelSat01-36 and MelSat02-26 contrasts with the remaining satellitome and indicate that satDNA diversification in *M. elongatus* increases as their clusters become smaller and more dispersed. Fast satDNA turnover may be another possibility, since almost all satDNA content is non-homologous in *M. elongatus*. *M. macrocephalus* presents 17 satDNA superfamilies (Utsunomia et al., 2019), but for *M. elongatus*, on the other hand, we recovered only one superfamily, which also endorses its highly diversified satDNA collection.

Also, most satDNAs were not detectable by physical mapping in neither sex, as presented in our present analyses and previous FISH results (Crepaldi and Parise-Maltempi, 2020). This confirms the majority of the satellitome for *M. elongatus* is arranged in small arrays below the detection threshold of FISH, corroborating other studies (Ruiz-Ruano et al., 2016; Bardella et al., 2020; Ferretti et al., 2020). SatDNA abundance is prone to rapidly change due to molecular mechanisms, such as dispersion and amplification (Garrido-Ramos, 2017), and can result in rapid repatterning as they expand or decrease their arrays (Plohl et al., 2008; Palacios-Gimenez et al., 2020; Sproul et al., 2020; Thakur et al., 2021) and this certainly is the case for *M. elongatus* as well.

This is a rather peculiar satellitome, especially considering our sex-biased analysis. Given the extraordinary heterochromatin content present in the female (owing to the heteromorphic W_1_ sex chromosome) (Galetti et al., 1995), it was initially assumed by us that the largest set of satDNAs would be female-biased as previously observed in *M. macrocephalus* (Utsunomia et al., 2019); however, not many satDNAs (in terms of number of different families) accounted for female-biased or W_1_-specific sequences in *M. elongatus.*

In what regards the male genome, satDNAs are equally abundant in qualitative terms; however, they are most likely becoming gradually lost and/or constrained instead of actually accumulating. Their low copies in the genome increase the possibility to escape homogenization mechanisms (Elder and Turner, 1995; Ugarković and Plohl, 2002; Plohl et al., 2012) as we noticed different homogenization patterns between the sexes. Repeats in larger arrays (and clustered in the W_1_ chromosome) had higher homogenization rates, and low abundance sequences (especially the male-biased ones) presented a more divergent pattern. It is possible to assume some non-exclusive explanations for this scenario. Firstly, more heterogeneity can be expected among repeating units if the mutation rate is high, relative to the rate in which a variant spreads through an array (Lorite et al., 2004; Ruiz-Ruano et al., 2019). Also, the heteromorphic W_1_ chromosome has a tendency to accumulate satDNAs already present in the female genome (Molina and Galetti, 2007; Crepaldi and Parise-Maltempi, 2020), which results in a quick, massive amplification in this sex in comparison to the poor clusterisation in the male counterpart (cf. Lower et al., 2018; Vondrak et al., 2020). In conclusion, satellitome diversity does not seem to be the main factor leading to W_1_ heterochromatic expansion in *M. elongatus*, instead the high amplification and homogenization rates of few particularly abundant satDNAs in the female genome effectively contributed to heterogametic sex chromosome differentiation.

Our second approach regarded a more evolutionary focus in the sex chromosome system and its satDNA content in *Megaleporinus elongatus,* as sex chromosome dynamics analysed through sex-linked repetitive DNA profiles has been shown to be effective (Traut and Winking, 2001; Schemberger et al., 2011; Cioffi et al., 2012; Terencio et al., 2012; Yano et al., 2016; Yano et al., 2017; Sember et al., 2018; Deakin et al., 2019; Schemberger et al., 2019). The heteromorphic sex chromosomes (W and W_1_) in *Megaleporinus* have already been deemed derived from a common ancestral chromosomic pair and differentiated from the Z chromosomes via massive heterochromatinization (Galetti and Foresti, 1986; Marreta et al., 2012; Parise-Maltempi et al., 2013; de Barros et al., 2018). However, variation in repetitive content within the genus could be caused by the expansion and contraction of these sequences (Utsunomia et al., 2019; Crepaldi and Parise-Maltempi, 2020).

Regardless of the library size of satDNAs, usually one or very few satDNA families are the most predominant in each species (Ruiz-Ruano et al., 2016; Silva et al., 2017; Bardella et al., 2020; da Silva et al., 2020) and the best candidates were none other but the two most abundant satDNA in *M. elongatus*, MelSat01-36 and MelSat02-26. Both satDNAs have concomitant origins in both sexes of *M. elongatus* and *M. macrocephalus,* but they have different evolutionary pathways in each species and concerted evolution might be acting separately for each of them.

MelSat01-36 is shared by W and W_1_ *,* but it is clustered only in males of one species (with prominent FISH signals being present in *M. macrocephalus* but absent in *M. elongatus*). Its two distinct clusters in female *M. elongatus* prompted us to combine all of our data on this satDNA and attempt to trace its relatedness between the species. All individuals presented similarly low divergence values for this satDNA, indicating a recent amplification for both species. Although identical sequences for MelSat01-36 are shared between the species, copy number can vary dramatically even among related organisms, without necessarily varying in their nucleotide sequences (Ugarković and Plohl, 2002; Louzada et al., 2020). Given our MST for this satDNA (Figure 2), ancestral species for *M. elongatus* and *M. macrocephalus* most likely presented the same conformation as *M. macrocephalus,* with one cluster in the ancestral W sex chromosome in the female and another cluster in a male autosomal chromosome. For *M. elongatus*, the male has lower copy numbers and a dispersed organization, since it did not present any FISH signals. This loss of autosomal clusters might have contributed to the homogenization in W_1_ in *M. elongatus,* and generated the enriched and duplicated regions in this chromosome, fixating in the female genome as described for many other organisms (Dalíková et al., 2017; Palacios-Gimenez et al., 2017; da Silva et al., 2020; Ferretti et al., 2020; Rovatsos et al., 2021).

MelSat02-26, on the other hand, is an older satDNA, with higher divergence values for both species and it is practically absent in male *M. macrocephalus*. Despite shared between the heteromorphic W and W_1_, it has a much less conserved pattern (Figure 2) in comparison to MelSat01-36, with dispersed clusters and almost none shared haplotypes between the females. An ancestral library for this satDNA cluster was most likely present in both the autosomes and the heteromorphic chromosome, much like MelSat01-36, however remaining highly amplified on both sexes of *M. elongatus.* It most likely had more time to diverge between the species and fixate in *M. elongatus* as well-defined clusters, as it is also co-localized in the putative multiple sex chromosomes (Z_2_ and W_2_). What previously seemed like having different accumulation in terms of time for each chromosome (Crepaldi and Parise-Maltempi, 2020), now, with more intimate evolutionary details, we can see that MelSat02-26 seems to have emerged equally in all *M. elongatus* chromosomes; along with its exclusive clusterisation pattern in the female Z_2_ and W_2_, shared with no other autosomal satDNA in the species and very particular to this sex.

The dynamics of the most abundant satDNAs in *M. elongatus* demonstrates not only their intimate evolution with the highly differentiated heteromorphic W_1_ sex chromosome, but also confirms that not only general molecular satDNA evolution is at play; particular characteristics of the species also concomitantly generate diversified satellitomes (Ugarković and Plohl, 2002; Lorite et al., 2004, Lorite et al., 2017; Richard et al., 2008; Palacios-Gimenez et al., 2017; Cabral-de-Mello et al., 2021). The ZW-based multiple sex chromosome system in *M. elongatus* shares a conserved heteromorphic W chromosome with the remaining *Megaleporinus*; but as seen by the two most prevalent satDNAs in the species their interspecific differences show distinct paces for W/W_1_ differentiation.

SatDNA evolution seems to be not only recent but very fast-paced in the genus, with high sequence turnover rates, and this might contribute to an equally fast and independent differentiation process in their young sex chromosome systems. A clear outcome of this is the highly amplified MelSat02-26 in Z_2_ and W_2_ in *M. elongatus* female, an interesting satDNA on its own as it partially represents a bigger repetitive sequence and molecular marker (Le*Spe*I) for the putative multiple chromosomes (Parise-Maltempi et al., 2007; Crepaldi and Parise-Maltempi, 2020). While MelSat02-26 is underrepresented in the female Z_1_ chromosome, the unusual female-specific clustered pattern shared by W1 and the novel elements (W_2_ and Z_2_) might indicate its spread via ectopic recombination, which has most likely helped this satDNA expand into large, homogeneous arrays (Ugarković and Plohl, 2002; Lower et al., 2018).

The rare Z_1_Z_1_Z_2_Z_2_/Z_1_W_1_Z_2_W_2_ system of *M. elongatus* is so far only found in one other fish species (*Ancistrus dolichopterus*) (de Oliveira et al., 2008; Favarato et al., 2016), and remains a puzzling occurrence even among multiple sex chromosome systems as whole (Sember et al., 2021). Analyzing other *Megaleporinus* as well as the sister genus *Leporinus* (comprising species without heteromorphic sex chromosomes) will eventually broaden our understanding of satDNA diversity and sex chromosome evolution as a whole in this group.

In the present study, we used cytogenomic approaches for satDNA analysis and visualization of their male and female heterogeneity. The results corroborate the recent spike of highly clustered repetitive content in the female genome and showed it has very distinctive characteristics even in comparison to closely-related species. Also, the recent burst of repetitive satDNA in the multiple sex chromosome system is still an ongoing process under the molecular mechanisms for satDNA evolution.

## Data Availability

The datasets presented in this study can be found in online repositories and accession number(s) can be found in the article.
