# Supplementary material for: Genomic Differences Between the Sexes in a Fish Species Seen Through Satellite DNAs
Source: Front Genet. 2021 Sep 30;12:728670. doi: 10.3389/fgene.2021.728670 (PMC8514694; doi:10.3389/fgene.2021.728670)
Supplement: Supplementary file 1 [file Image5.pdf]

Supplementary Figure S5. Individual line color-coded landscapes (abundance in the genome vs Kimura distance-based copy divergence) for each selected female-biased satDNAs. The line plots are arranged in decreasing order according to the F/M ratio quotients (Supplementary Table S2).

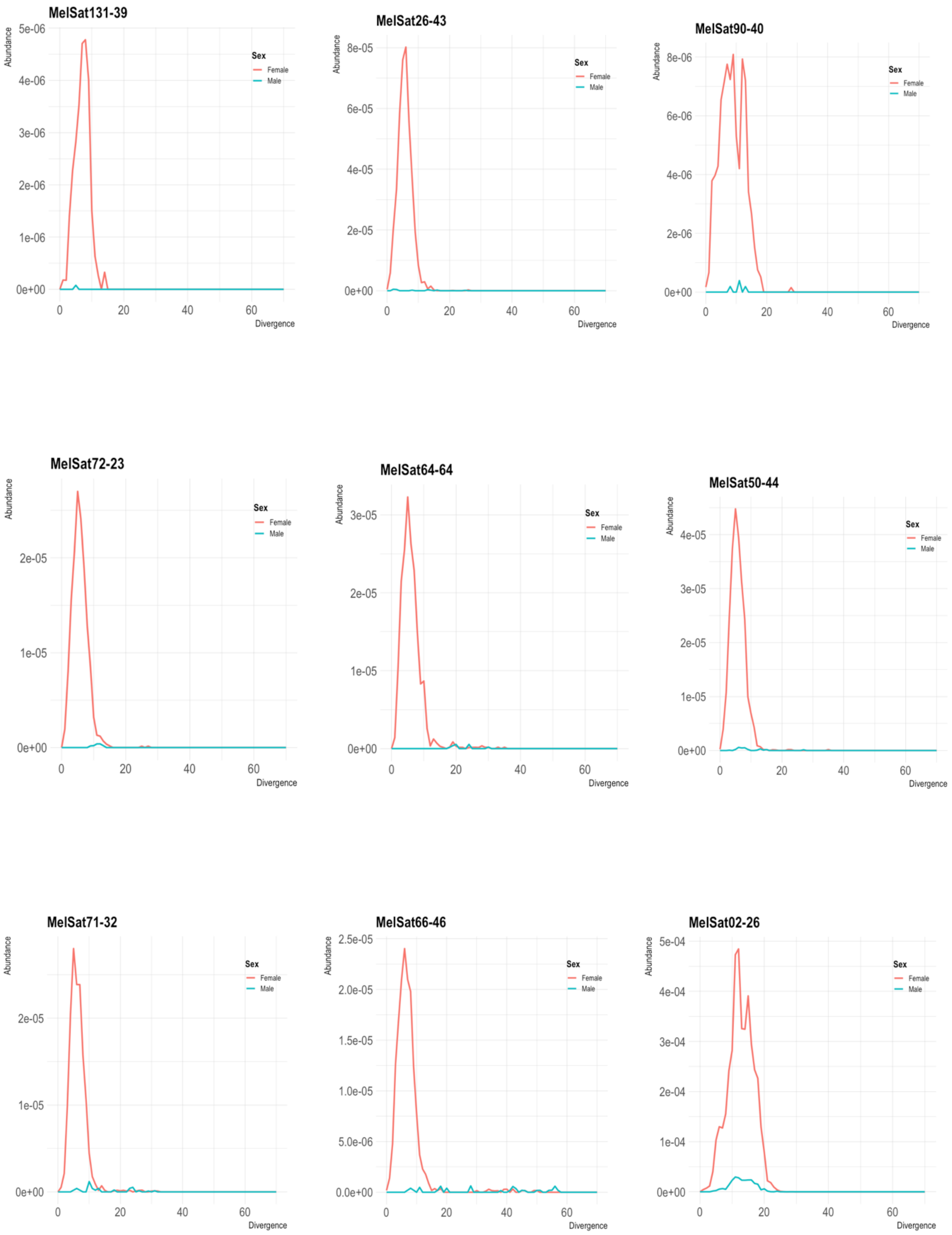

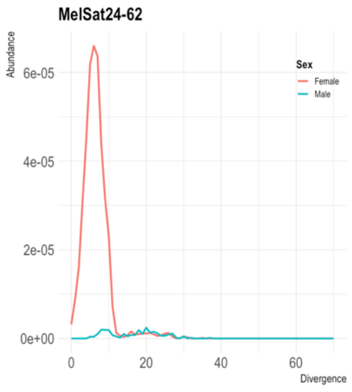
MelSat24-62
Abundance
6e-05
4e-05
2e-05
0e+00
0
20
40
60
Divergence
Sex
Female
Male

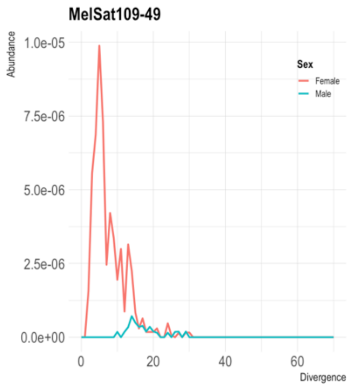
MelSat109-49
Abundance
1.0e-05
7.5e-06
5.0e-06
2.5e-06
0.0e+00
0
20
40
60
Divergence
Sex
Female
Male

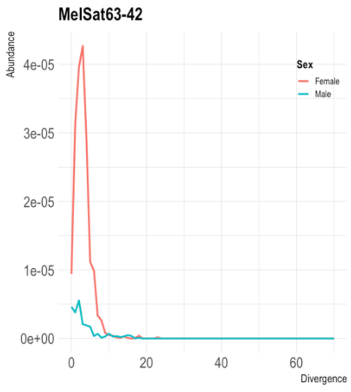
MelSat63-42
Abundance
4e-05
3e-05
2e-05
1e-05
0e+00
0
20
40
60
Divergence
Sex
Female
Male

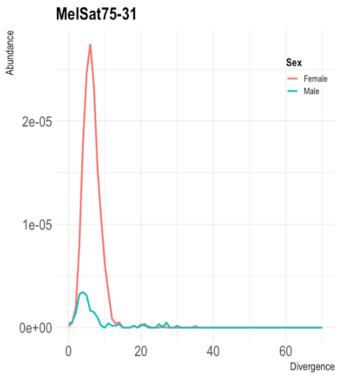
MelSat75-31
Abundance
2e-05
1e-05
0e+00
0
20
40
60
Divergence
Sex
Female
Male

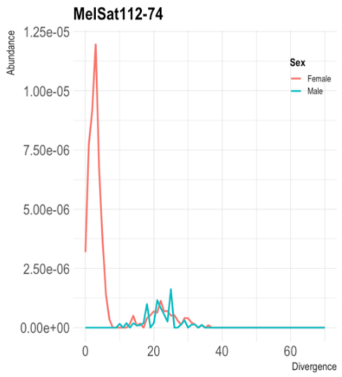
MelSat112-74
Abundance
1.25e-05
1.00e-05
7.50e-06
5.00e-06
2.50e-06
0.00e+00
0
20
40
60
Divergence
Sex
Female
Male

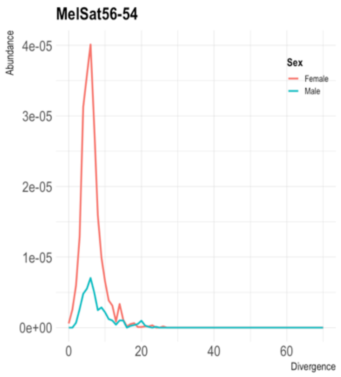
MelSat56-54
Abundance
4e-05
3e-05
2e-05
1e-05
0e+00
0
20
40
60
Divergence
Sex
Female
Male

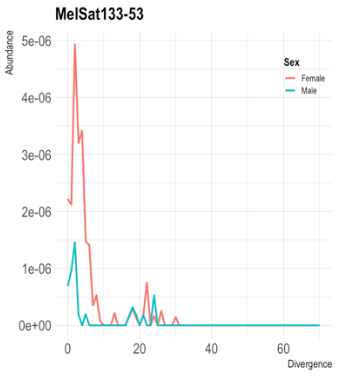
MelSat133-53
Abundance
5e-06
4e-06
3e-06
2e-06
1e-06
0e+00
0
20
40
60
Divergence
Sex
Female
Male

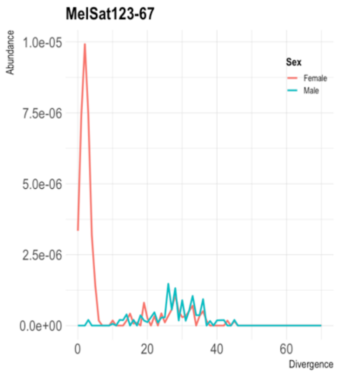
MelSat123-67
Abundance
1.0e-05
7.5e-06
5.0e-06
2.5e-06
0.0e+00
0
20
40
60
Divergence
Sex
Female
Male

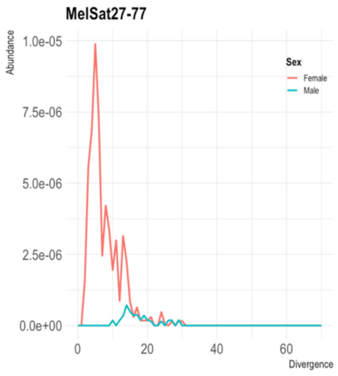
MelSat27-77
Abundance
1.0e-05
7.5e-06
5.0e-06
2.5e-06
0.0e+00
0
20
40
60
Divergence
Sex
Female
Male

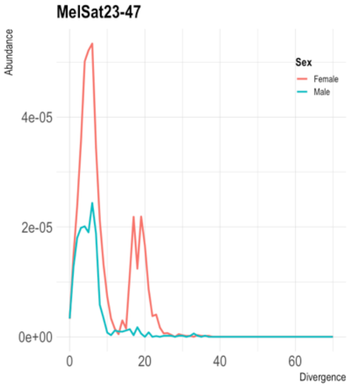
MelSat23-47
Abundance
4e-05
2e-05
0e+00
0
20
40
60
Divergence
Sex
Female
Male

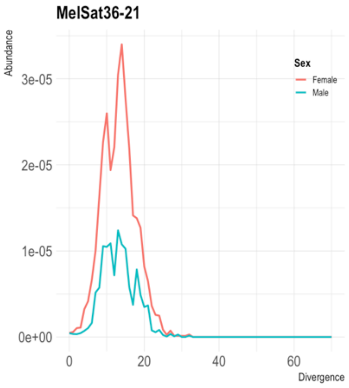
MelSat36-21
Abundance
3e-05
2e-05
1e-05
0e+00
0
20
40
60
Divergence
Sex
Female
Male

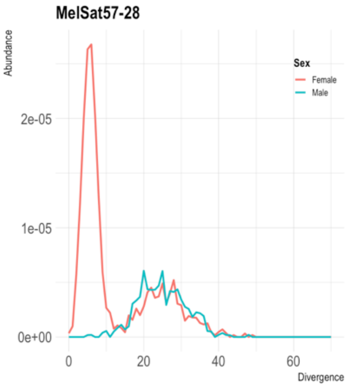
MelSat57-28
Abundance
2e-05
1e-05
0e+00
0
20
40
60
Divergence
Sex
Female
Male

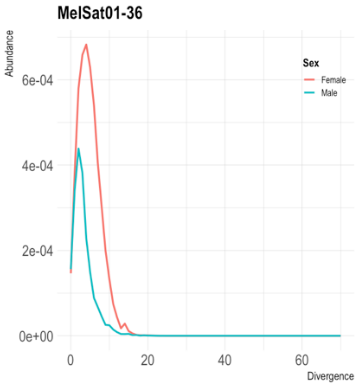
MelSat01-36
Abundance
6e-04
4e-04
2e-04
0e+00
0
20
40
60
Divergence
Sex
Female
Male
