# Supplementary material for: Genomic Differences Between the Sexes in a Fish Species Seen Through Satellite DNAs
Source: Front Genet. 2021 Sep 30;12:728670. doi: 10.3389/fgene.2021.728670 (PMC8514694; doi:10.3389/fgene.2021.728670)
Supplement: Supplementary file 2 [file Table1.pdf]

Supplementary Table S1. General characteristics of satDNA families recovered from male and female genomes of *M. elongatus*, such as number of variants, length (bp), A+T (%), female (F) and male (M) abundance (% of the genome) and female (F) and male (M) divergence (%). Each satDNA has their own quotient for female and male abundance (F/M ratio). The only superfamily (SF) is also evidenced.

SF	satDNA	Variants	Length	A+T	Abundance		Divergence		F/M
					F	M	F	M	
	MelSat01-36	1	36	66.7	0.4844	0.1991	5.02	3.35	2.43
	MelSat02-26	3	26	30.8	0.4130	0.0253	13.16	13.54	16.31
	MelSat03-177	2	177	66.6	0.3384	0.3218	9.1	9.22	1.05
	MelSat04-24	7	24	62.5	0.1739	0.2371	6.91	6.31	0.73
	MelSat05-21	5	21	76.2	0.1610	0.1579	18.72	19.09	1.02
	MelSat06-51	1	51	80.4	0.1548	0.1276	4.16	4.38	1.21
	MelSat07-37	2	37	56.7	0.1520	0.0982	5.49	3.59	1.55
	MelSat08-42	10	42	57.2	0.1386	0.1920	13.83	13.24	0.72
1	MelSat09-60	2	60	61.6	0.1231	0.1220	20.57	21.1	1.01
	MelSat10-61	2	61	72.1	0.1168	0.1063	3.06	3.19	1.10
	MelSat11-245	1	245	60.4	0.1158	0.1393	10.41	10.16	0.83
	MelSat12-67	4	67	62.7	0.1062	0.0599	9.72	10.66	1.77
	MelSat13-20	2	20	50	0.1011	0.1347	5.83	4.15	0.75
	MelSat14-52	3	52	53.9	0.0852	0.1112	10.27	10.23	0.77
	MelSat15-42	2	42	56.1	0.0672	0.0607	8	7	1.11
	MelSat16-25	1	25	60	0.0618	0.0332	9.13	7.82	1.86
	MelSat17-35	1	35	57.2	0.0612	0.0256	14.13	11	2.39
	MelSat18-67	1	67	53.8	0.0594	0.0626	10.04	9.65	0.95
	MelSat19-73	2	73	68.5	0.0517	0.0459	14.85	14.89	1.12
	MelSat20-53	2	53	58.4	0.0497	0.0555	7.87	7.29	0.90
	MelSat21-41	5	41	61	0.0467	0.0422	8.25	8.48	1.11
	MelSat22-34	1	34	64.8	0.0425	0.0379	6.07	6.19	1.12

MelSat23-47	1	47	57.4	0.0425	0.0158	9.05	5.68	2.69
MelSat24-62	1	62	71	0.0417	0.0026	6.81	17.31	16.25
MelSat25-30	13	30	53.4	0.0415	0.0366	13.41	14.51	1.13
MelSat26-43	1	43	62.8	0.0403	0.0002	5.92	9.12	212.94
MelSat27-77	2	77	62.4	0.0399	0.0123	8.2	11.73	3.24
MelSat28-35	2	35	55.9	0.0393	0.0445	14.92	14.85	0.88
MelSat29-121	1	121	50.4	0.0371	0.0255	6.58	6.56	1.46
MelSat30-22	1	22	59.1	0.0353	0.0626	6.54	6.09	0.56
MelSat31-72	1	72	58.4	0.0352	0.0157	4.88	5.56	2.24
MelSat32-17	11	17	53	0.0349	0.0262	10.15	10.9	1.33
MelSat33-37	5	37	56.7	0.0333	0.0383	8.62	9.76	0.87
MelSat34-41	1	41	68.3	0.0319	0.0228	7.86	8.62	1.40
MelSat35-58	1	58	67.2	0.0318	0.0350	4.47	4.73	0.91
MelSat36-21	2	21	57.1	0.0314	0.0121	13.54	13.31	2.60
MelSat37-90	1	90	70	0.0311	0.0335	3.73	3.66	0.93
MelSat38-21	9	21	71.5	0.0302	0.0348	23.94	22.75	0.87
MelSat39-65	1	65	64.6	0.0300	0.0311	7.56	7.65	0.96
MelSat40-52	2	52	65.4	0.0293	0.0277	14.61	14.31	1.06
MelSat41-35	3	35	51.5	0.0275	0.0305	11.01	10.83	0.90
MelSat42-29	6	29	79.3	0.0263	0.0243	17.4	17.42	1.08
MelSat43-26	1	26	76.9	0.0262	0.1059	7.26	6.81	0.25
MelSat44-52	5	52	65.4	0.0258	0.0359	6.35	6.23	0.72
MelSat45-31	5	31	51.6	0.0251	0.0354	16.62	16.4	0.71
MelSat46-45	1	45	53.3	0.0248	0.0516	6.07	4.9	0.48
MelSat47-39	2	39	53.9	0.0246	0.0264	12.46	11.68	0.93
MelSat48-29	2	29	51.7	0.0243	0.0382	16.37	14.44	0.64
MelSat49-33	2	33	60.6	0.0241	0.0571	7.03	7.53	0.42

MelSat50-44	1	44	59.1	0.0240	0.0003	6	9.79	87.02
MelSat51-29	2	29	55.1	0.0237	0.0251	29.13	28.75	0.95
MelSat52-38	2	38	50	0.0224	0.0283	11.09	10.92	0.79
MelSat53-42	1	42	61.9	0.0212	0.0098	8.29	6.5	2.16
MelSat54-30	3	30	63.3	0.0209	0.0246	14.01	14.07	0.85
MelSat55-21	3	21	65	0.0208	0.0114	19.6	20.26	1.83
MelSat56-54	1	54	61.1	0.0205	0.0041	6.52	8.08	5.05
MelSat57-28	2	28	60.7	0.0203	0.0080	12.61	25.38	2.54
MelSat58-31	6	31	61.3	0.0202	0.0302	20.09	17.91	0.67
MelSat59-61	2	61	50.8	0.0197	0.0962	7.77	7.43	0.20
MelSat60-48	3	48	64.6	0.0196	0.0149	8.69	9.02	1.32
MelSat61-21	3	21	65	0.0187	0.0185	14.32	14.8	1.01
MelSat62-32	6	32	50	0.0187	0.0321	11.86	10.74	0.58
MelSat63-42	1	42	64.3	0.0182	0.0024	3.32	3.78	7.61
MelSat64-64	1	64	73.4	0.0181	0.0002	6.34	22.44	98.34
MelSat65-28	1	28	75	0.0158	0.0217	8.88	8.63	0.73
MelSat66-46	2	46	58.7	0.0154	0.0006	7.38	33.02	24.73
MelSat67-31	1	31	45.2	0.0152	0.0215	10.9	10.08	0.70
MelSat68-31	2	31	61.3	0.0151	0.0146	12.93	11.81	1.03
MelSat69-33	1	33	57.5	0.0149	0.0287	4.68	4.15	0.52
MelSat70-54	2	54	46.3	0.0146	0.0141	14.5	14.91	1.03
MelSat71-32	1	32	68.7	0.0145	0.0004	6.62	14.95	32.34
MelSat72-23	2	23	78.2	0.0145	0.0001	5.94	11.61	107.76
MelSat73-50	4	50	64	0.0144	0.0138	10.76	10.06	1.04
MelSat74-67	1	67	68.6	0.0143	0.0163	6.6	6.93	0.88
MelSat75-31	1	31	61.3	0.0142	0.0019	6.79	6.89	7.37
MelSat76-42	4	42	62	0.0141	0.0146	17.46	17.27	0.97

MelSat77-17	1	17	58.8	0.0141	0.0166	12.86	11.22	0.85
MelSat78-24	2	24	50	0.0137	0.0237	21.86	20.15	0.58
MelSat79-21	2	21	71.4	0.0128	0.0124	24.34	24.14	1.04
MelSat80-50	2	50	68	0.0125	0.0307	5.4	5.21	0.41
MelSat81-49	1	49	79.6	0.0122	0.0146	8.32	8.21	0.84
MelSat82-21	2	21	47.6	0.0102	0.0124	20.68	20.34	0.82
MelSat83-26	1	26	65.4	0.0095	0.0124	10.14	9.88	0.76
MelSat84-41	4	41	53.7	0.0093	0.0117	17.84	17.05	0.79
MelSat85-31	11	31	67.7	0.0092	0.0100	14.47	14.24	0.91
MelSat86-21	1	21	53.2	0.0090	0.0106	12.46	11.86	0.84
MelSat87-17	3	17	58.8	0.0089	0.0119	9.88	8.96	0.75
MelSat88-30	1	30	50	0.0088	0.0107	7.87	7.29	0.82
MelSat89-46	2	46	54.4	0.0086	0.0036	8.31	9.68	2.40
MelSat90-40	1	40	60	0.0083	0.0001	9.11	11.36	108.09
MelSat91-30	1	30	63.3	0.0083	0.0118	6.79	6.74	0.71
MelSat92-31	2	31	55.1	0.0082	0.0146	8.58	7.76	0.56
MelSat93-27	1	27	48.1	0.0079	0.0071	6.92	6.06	1.12
MelSat94-41	7	41	58.5	0.0078	0.0108	15.71	13.69	0.72
MelSat95-30	1	30	66.7	0.0075	0.0044	13.53	13.62	1.71
MelSat96-48	1	48	48	0.0068	0.0103	6.49	5.58	0.66
MelSat97-61	1	61	62.3	0.0065	0.0079	5.95	5.76	0.82
MelSat98-69	1	69	68.1	0.0065	0.0169	3.9	3.9	0.39
MelSat99-44	1	44	61.3	0.0064	0.0079	12.64	10.53	0.81
MelSat100-22	1	22	63.7	0.0063	0.0116	8.22	7.64	0.54
MelSat101-15	3	15	60	0.0063	0.0114	15.4	14.91	0.55
MelSat102-38	1	38	55.3	0.0062	0.0150	3.41	2.75	0.42
MelSat103-67	1	67	62.7	0.0061	0.0085	7.91	8.12	0.73

	MelSat104-57	1	57	54.4	0.0060	0.0065	9.11	8.77	0.92
	MelSat105-31	1	31	54.8	0.0059	0.0059	8.13	8.42	1.00
	MelSat106-60	3	60	56.7	0.0058	0.0089	9.09	8.41	0.65
	MelSat107-11	2	11	72.8	0.0057	0.0057	12.74	12.77	1.01
	MelSat108-48	2	48	72.9	0.0057	0.0072	14.6	13.93	0.79
	MelSat109-49	1	49	63.3	0.0056	0.0004	7.94	17.96	13.22
	MelSat110-32	1	32	56.3	0.0056	0.0075	8.92	8.82	0.74
	MelSat111-19	1	19	57.9	0.0055	0.0031	7.04	7.58	1.76
	MelSat112-74	1	74	62.2	0.0053	0.0007	6.05	22.45	7.14
1	MelSat113-60	1	60	60	0.0053	0.0055	17.33	16.45	0.96
	MelSat114-21	3	21	66.7	0.0052	0.0080	11.27	11.83	0.64
	MelSat115-55	1	55	56.3	0.0050	0.0049	8.11	8.17	1.02
	MelSat116-37	1	37	59.4	0.0049	0.0076	14.85	14.25	0.65
	MelSat117-49	1	49	51	0.0048	0.0085	5.41	4.58	0.57
	MelSat118-43	1	43	58.1	0.0046	0.0073	25.82	16.21	0.63
	MelSat119-62	1	62	61.3	0.0045	0.0045	11.55	12.09	1.00
	MelSat120-29	1	29	44.8	0.0045	0.0048	8.55	8.27	0.94
	MelSat121-18	2	18	58.8	0.0044	0.0061	11.32	8.79	0.71
	MelSat122-40	2	40	77.5	0.0043	0.0053	5.38	4.9	0.81
	MelSat123-67	1	67	64.1	0.0041	0.0012	7.45	28.22	3.44
	MelSat124-53	1	53	58.5	0.0041	0.0252	7.11	6.27	0.16
	MelSat125-31	2	31	61.3	0.0039	0.0029	10.61	16.36	1.37
	MelSat126-28	1	28	53.6	0.0038	0.0040	18.86	19.23	0.96
	MelSat127-29	4	29	51.7	0.0033	0.0039	14.23	13.59	0.85
	MelSat128-28	1	28	64.3	0.0032	0.0037	2.93	2.84	0.88
	MelSat129-45	2	45	64.5	0.0030	0.0064	9.5	9.41	0.47
	MelSat130-56	1	56	57.1	0.0028	0.0029	4.24	3.48	0.95

MelSat131-39	1	39	53.9	0.0027	0.0000	7.34	5.32	347.44
MelSat132-36	1	36	52.7	0.0023	0.0045	5.49	4.74	0.50
MelSat133-53	1	53	50.9	0.0022	0.0005	4.93	7.19	4.52
MelSat134-17	1	17	58.8	0.0022	0.0019	14.27	11.35	1.14
MelSat135-66	1	66	69.7	0.0020	0.0034	6.2	4.44	0.60
MelSat136-31	1	31	54.8	0.0017	0.0020	12.16	14.41	0.85
MelSat137-21	1	21	57.1	0.0016	0.0026	16.08	14.23	0.60
MelSat138-35	1	35	54.3	0.0010	0.0009	9.92	9.67	1.08
MelSat139-57	1	57	33.3	0.0006	0.0007	15.25	19.94	0.97
MelSat140-24	1	24	58.3	0.0005	0.0010	6	10.1	0.50
