# Supplementary material for: Genomic Differences Between the Sexes in a Fish Species Seen Through Satellite DNAs
Source: Front Genet. 2021 Sep 30;12:728670. doi: 10.3389/fgene.2021.728670 (PMC8514694; doi:10.3389/fgene.2021.728670)
Supplement: Supplementary file 3 [file Table2.pdf]

Supplementary Table S2. Female to male ratio for all isolated satDNAs of *M. elongatus*, arranged by most female-biased families (top of the table, ratio higher than 1) to most male-biased ones (bottom of the table, ratio below 1) and their respective abundance (%) in both genomes, female (F) and male (M).

satDNA	Abundance		F/M
	F	M	
MelSat131-39	0.0027	0.0000	347.44
MelSat26-43	0.0403	0.0002	212.94
MelSat90-40	0.0083	0.0001	108.09
MelSat72-23	0.0145	0.0001	107.76
MelSat64-64	0.0181	0.0002	98.34
MelSat50-44	0.0240	0.0003	87.02
MelSat71-32	0.0145	0.0004	32.34
MelSat66-46	0.0154	0.0006	24.73
MelSat02-26	0.4130	0.0253	16.31
MelSat24-62	0.0417	0.0026	16.25
MelSat109-49	0.0056	0.0004	13.22
MelSat63-42	0.0182	0.0024	7.61
MelSat75-31	0.0142	0.0019	7.37
MelSat112-74	0.0053	0.0007	7.14
MelSat56-54	0.0205	0.0041	5.05
MelSat133-53	0.0022	0.0005	4.52
MelSat123-67	0.0041	0.0012	3.44
MelSat27-77	0.0399	0.0123	3.24
MelSat23-47	0.0425	0.0158	2.69
MelSat36-21	0.0314	0.0121	2.60
MelSat57-28	0.0203	0.0080	2.54
MelSat01-36	0.4844	0.1991	2.43
MelSat89-46	0.0086	0.0036	2.40

MelSat17-35	0.0612	0.0256	2.39
MelSat31-72	0.0352	0.0157	2.24
MelSat53-42	0.0212	0.0098	2.16
MelSat16-25	0.0618	0.0332	1.86
MelSat55-21	0.0208	0.0114	1.83
MelSat12-67	0.1062	0.0599	1.77
MelSat111-19	0.0055	0.0031	1.76
MelSat95-30	0.0075	0.0044	1.71
MelSat07-37	0.1520	0.0982	1.55
MelSat29-121	0.0371	0.0255	1.46
MelSat34-41	0.0319	0.0228	1.40
MelSat125-31	0.0039	0.0029	1.37
MelSat32-17	0.0349	0.0262	1.33
MelSat60-48	0.0196	0.0149	1.32
MelSat06-51	0.1548	0.1276	1.21
MelSat134-17	0.0022	0.0019	1.14
MelSat25-30	0.0415	0.0366	1.13
MelSat19-73	0.0517	0.0459	1.12
MelSat22-34	0.0425	0.0379	1.12
MelSat93-27	0.0079	0.0071	1.12
MelSat15-42	0.0672	0.0607	1.11
MelSat21-41	0.0467	0.0422	1.11
MelSat10-61	0.1168	0.1063	1.10
MelSat42-29	0.0263	0.0243	1.08
MelSat138-35	0.0010	0.0009	1.08
MelSat40-52	0.0293	0.0277	1.06
MelSat03-177	0.3384	0.3218	1.05

MelSat73-50	0.0144	0.0138	1.04
MelSat79-21	0.0128	0.0124	1.04
MelSat68-31	0.0151	0.0146	1.03
MelSat70-54	0.0146	0.0141	1.03
MelSat05-21	0.1610	0.1579	1.02
MelSat115-55	0.0050	0.0049	1.02
MelSat61-21	0.0187	0.0185	1.01
MelSat107-11	0.0057	0.0057	1.01
MelSat09-60	0.1231	0.1220	1.01
MelSat119-62	0.0045	0.0045	1.00
MelSat105-31	0.0059	0.0059	1.00
MelSat139-57	0.0006	0.0007	0.97
MelSat76-42	0.0141	0.0146	0.97
MelSat39-65	0.0300	0.0311	0.96
MelSat113-60	0.0053	0.0055	0.96
MelSat126-28	0.0038	0.0040	0.96
MelSat130-56	0.0028	0.0029	0.95
MelSat18-67	0.0594	0.0626	0.95
MelSat51-29	0.0237	0.0251	0.95
MelSat120-29	0.0045	0.0048	0.94
MelSat47-39	0.0246	0.0264	0.93
MelSat37-90	0.0311	0.0335	0.93
MelSat104-57	0.0060	0.0065	0.92
MelSat85-31	0.0092	0.0100	0.91
MelSat35-58	0.0318	0.0350	0.91
MelSat41-35	0.0275	0.0305	0.90
MelSat20-53	0.0497	0.0555	0.90

MelSat28-35	0.0393	0.0445	0.88
MelSat128-28	0.0032	0.0037	0.88
MelSat74-67	0.0143	0.0163	0.88
MelSat33-37	0.0333	0.0383	0.87
MelSat38-21	0.0302	0.0348	0.87
MelSat54-30	0.0209	0.0246	0.85
MelSat127-29	0.0033	0.0039	0.85
MelSat136-31	0.0017	0.0020	0.85
MelSat77-17	0.0141	0.0166	0.85
MelSat86-21	0.0090	0.0106	0.84
MelSat81-49	0.0122	0.0146	0.84
MelSat11-245	0.1158	0.1393	0.83
MelSat97-61	0.0065	0.0079	0.82
MelSat82-21	0.0102	0.0124	0.82
MelSat88-30	0.0088	0.0107	0.82
MelSat99-44	0.0064	0.0079	0.81
MelSat122-40	0.0043	0.0053	0.81
MelSat108-48	0.0057	0.0072	0.79
MelSat84-41	0.0093	0.0117	0.79
MelSat52-38	0.0224	0.0283	0.79
MelSat14-52	0.0852	0.1112	0.77
MelSat83-26	0.0095	0.0124	0.76
MelSat13-20	0.1011	0.1347	0.75
MelSat87-17	0.0089	0.0119	0.75
MelSat110-32	0.0056	0.0075	0.74
MelSat04-24	0.1739	0.2371	0.73
MelSat65-28	0.0158	0.0217	0.73

MelSat103-67	0.0061	0.0085	0.73
MelSat08-42	0.1386	0.1920	0.72
MelSat94-41	0.0078	0.0108	0.72
MelSat44-52	0.0258	0.0359	0.72
MelSat121-18	0.0044	0.0061	0.71
MelSat45-31	0.0251	0.0354	0.71
MelSat91-30	0.0083	0.0118	0.71
MelSat67-31	0.0152	0.0215	0.70
MelSat58-31	0.0202	0.0302	0.67
MelSat96-48	0.0068	0.0103	0.66
MelSat106-60	0.0058	0.0089	0.65
MelSat116-37	0.0049	0.0076	0.65
MelSat114-21	0.0052	0.0080	0.64
MelSat48-29	0.0243	0.0382	0.64
MelSat118-43	0.0046	0.0073	0.63
MelSat135-66	0.0020	0.0034	0.60
MelSat137-21	0.0016	0.0026	0.60
MelSat62-32	0.0187	0.0321	0.58
MelSat78-24	0.0137	0.0237	0.58
MelSat117-49	0.0048	0.0085	0.57
MelSat30-22	0.0353	0.0626	0.56
MelSat92-31	0.0082	0.0146	0.56
MelSat101-15	0.0063	0.0114	0.55
MelSat100-22	0.0063	0.0116	0.54
MelSat69-33	0.0149	0.0287	0.52
MelSat132-36	0.0023	0.0045	0.50
MelSat140-24	0.0005	0.0010	0.50

MelSat46-45	0.0248	0.0516	0.48
MelSat129-45	0.0030	0.0064	0.47
MelSat49-33	0.0241	0.0571	0.42
MelSat102-38	0.0062	0.0150	0.42
MelSat80-50	0.0125	0.0307	0.41
MelSat98-69	0.0065	0.0169	0.39
MelSat43-26	0.0262	0.1059	0.25
MelSat59-61	0.0197	0.0962	0.20
MelSat124-53	0.0041	0.0252	0.16
