# Supplementary material for: Genomic Differences Between the Sexes in a Fish Species Seen Through Satellite DNAs
Source: Front Genet. 2021 Sep 30;12:728670. doi: 10.3389/fgene.2021.728670 (PMC8514694; doi:10.3389/fgene.2021.728670)

Supplementary Figure S4. Variant profiles for the 22 most female-biased satDNAs in decreasing order according to the ratio quotient for F/M ratio. Topmost profiles correspond to the female copies and bottom profiles to the male ones for each satDNA. Divergence and abundance values for each selected satDNA are also specified. F = female; M = male.

MelSat131-39
Divergence: F = 7.34% / M = 5.32%
Abundance: F = 0.0027% / M = 0.0000%
F/M ratio = 347.4

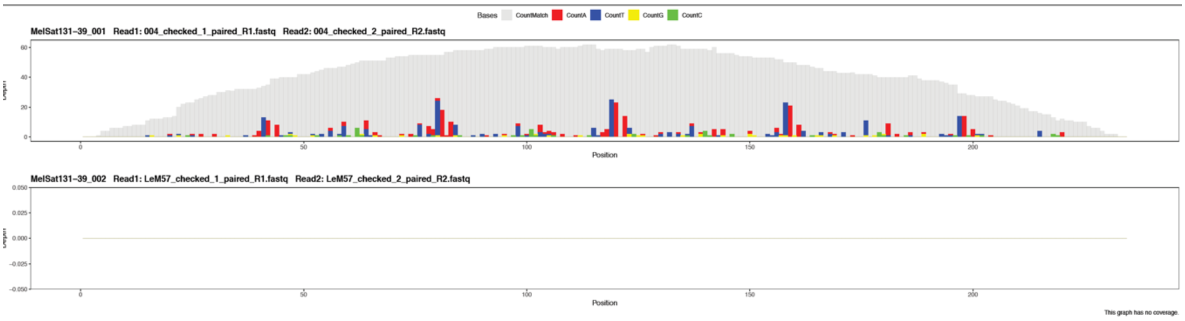

MelSat26-43
Divergence: F = 5.92% / M = 9.12%
Abundance: F = 0.0403% / M = 0.0002%
F/M ratio = 212.9

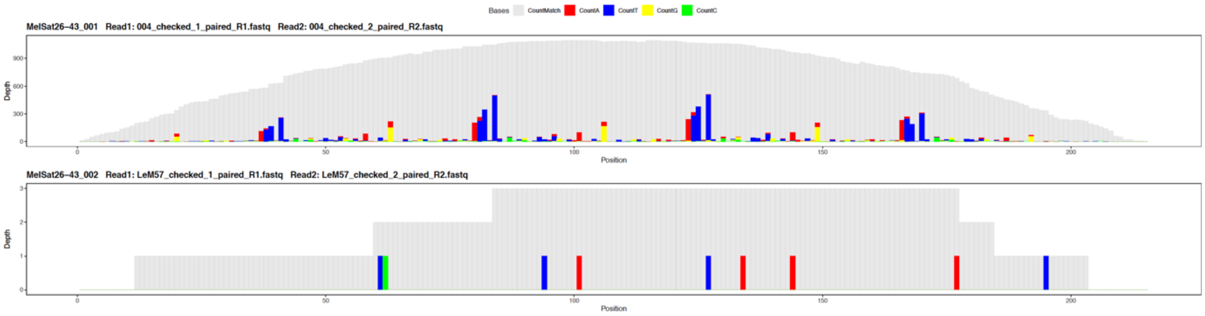

MelSat90-40
Divergence: F = 9.11% / M = 11.36%
Abundance: F = 0.0083% / M = 0.0001%
F/M ratio = 108.0

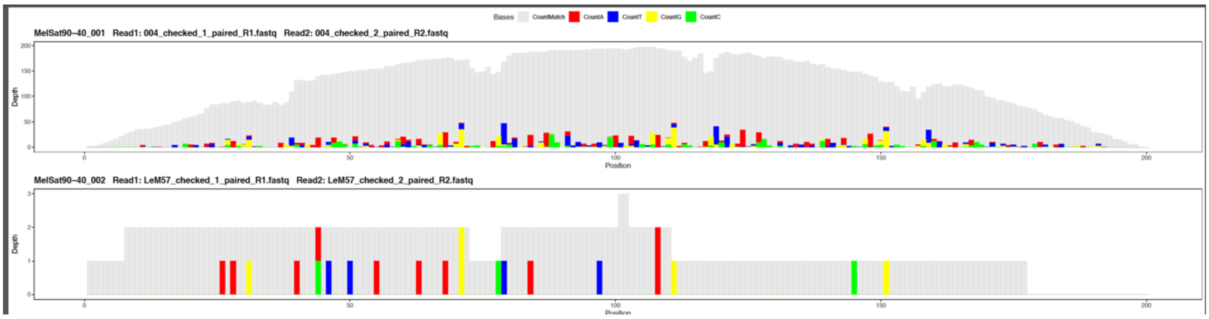

MelSat72-23
Divergence: F = 5.94% / M = 11.61%
Abundance: F = 0.0145% / M = 0.0001%
F/M ratio = 107.7

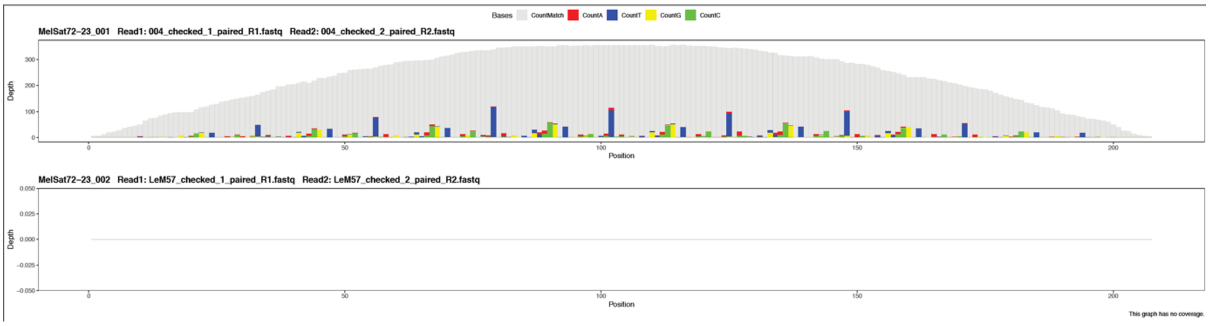

MelSat64-64
Divergence: F = 6.34% / M = 22.44%
Abundance: F = 0.0181% / M = 0.0002%
F/M ratio = 98.33

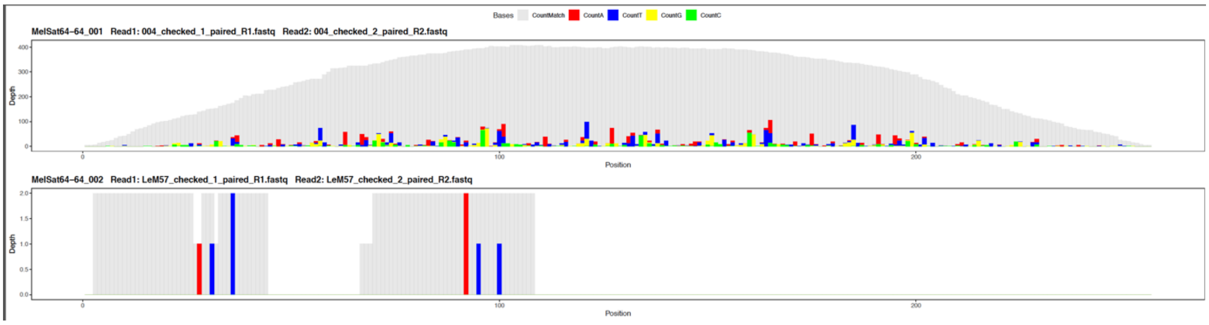

MelSat50-44
Divergence: F = 6% / M = 9.79%
Abundance: F = 0.0240% / M = 0.0003%
F/M ratio = 87.02

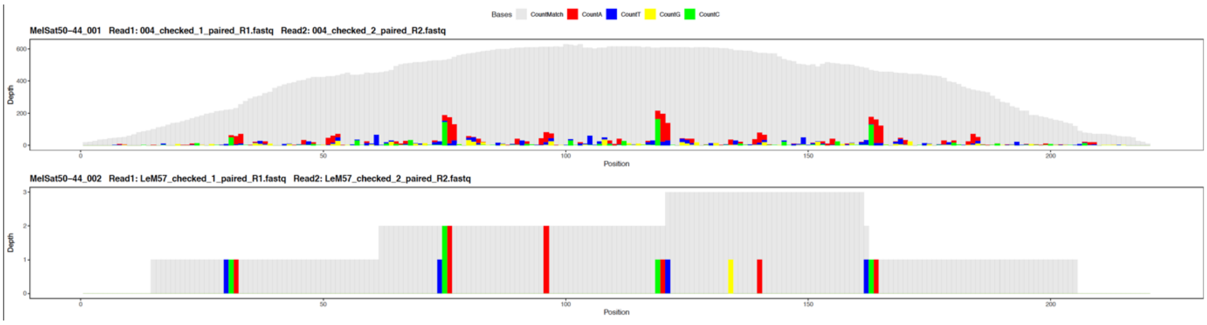

MelSat71-32
Divergence: F = 6.62% / M = 14.95%
Abundance: F = 0.0145% / M = 0.0004%
F/M ratio = 32.34

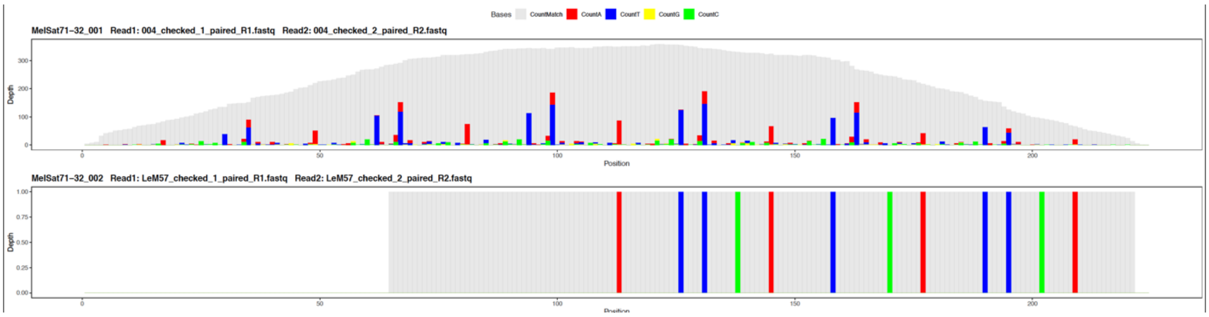

MelSat66-46
Divergence: F = 7.38% / M = 33%
Abundance: F = 0.0154% / M = 0.0006%
F/M ratio = 24.72

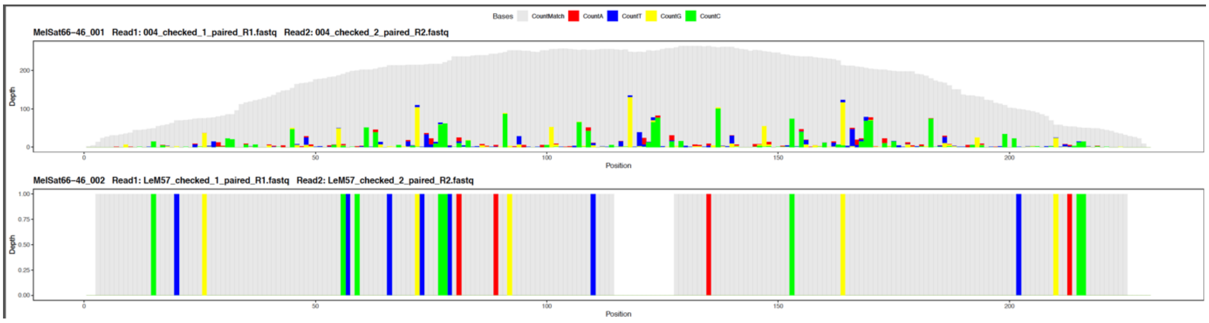

MelSat02-26
Divergence: F = 13.16% / M = 13.54%
Abundance: F = 0.4130% / M = 0.0253%
F/M ratio = 16.31

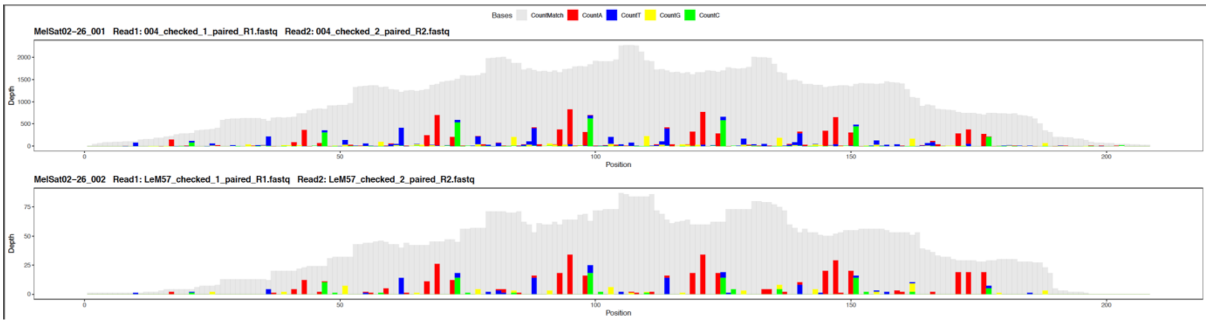

MelSat24-62
Divergence: F = 6.81% / M = 17.31%
Abundance: F = 0.0417% / M = 0.0026%
F/M ratio = 16.25

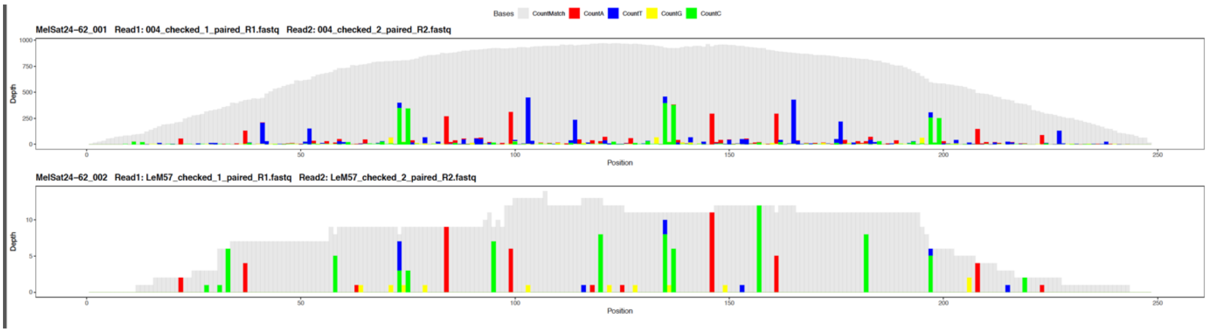

MelSat109-49
Divergence: F = 7.94% / M = 17.96%
Abundance: F = 0.0056% / M = 0.0004%
F/M ratio = 13.21

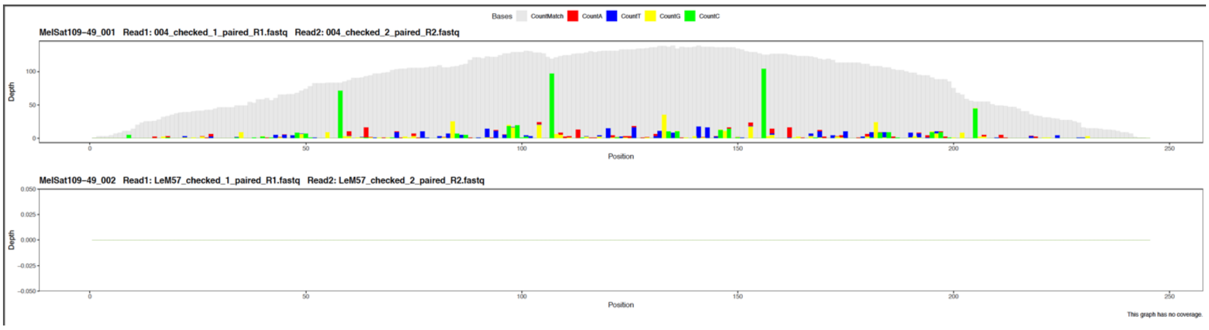

MelSat63-42
Divergence: F = 3.32% / M = 3.78%
Abundance: F = 0.0182% / M = 0.0024%
F/M ratio = 7.612

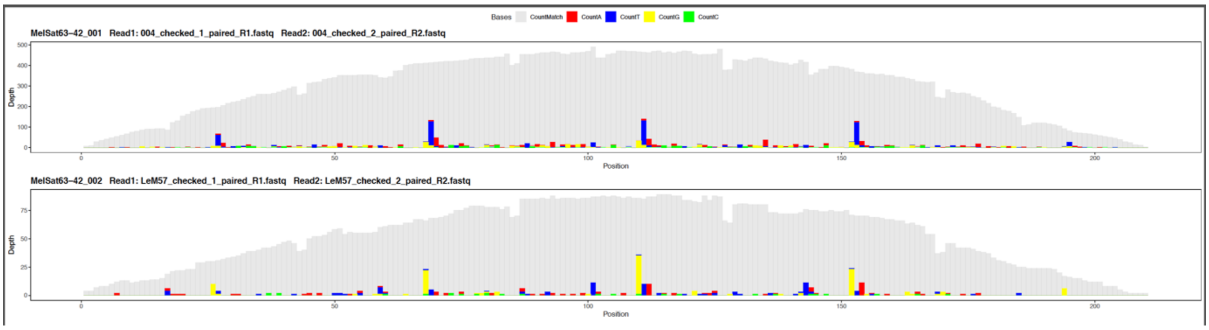

MelSat75-31
Divergence: F = 6.79% / M = 6.89%
Abundance: F = 0.0142% / M = 0.0019%
F/M ratio = 7.37

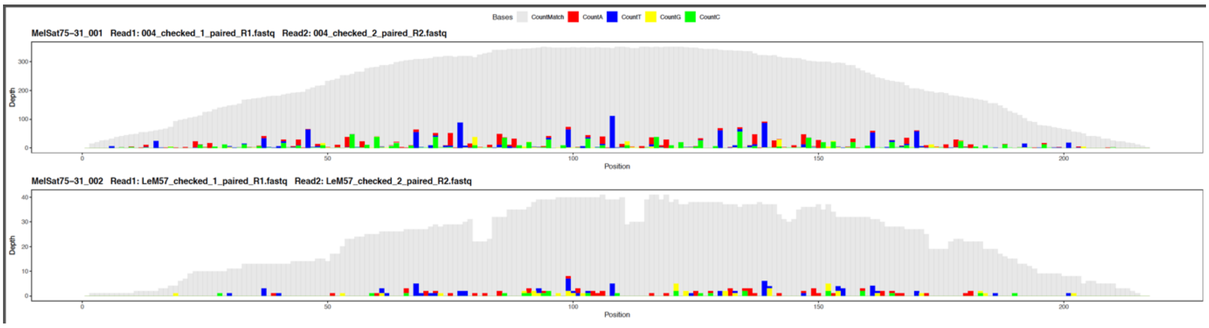

MelSat112-74
Divergence: F = 6.05% / M = 22.45%
Abundance: F = 0.0053% / M = 0.0007%
F/M ratio = 7.14

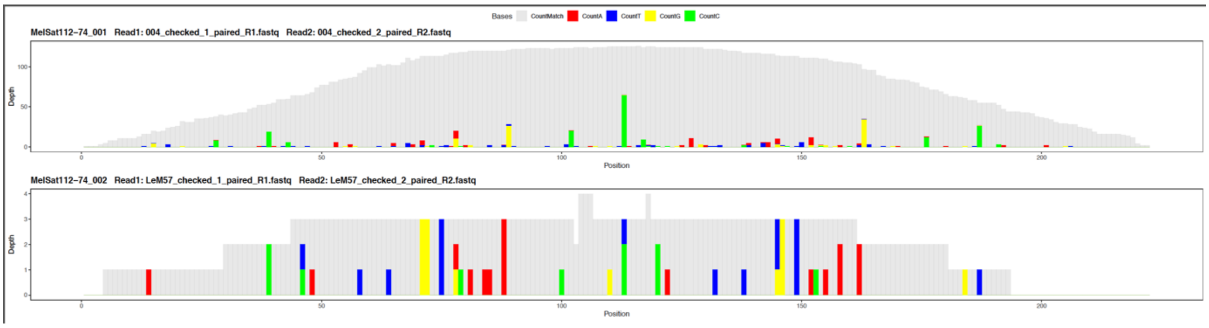

MelSat56-54
Divergence: F = 6.52% / M = 8%
Abundance: F = 0.0205% / M = 0.0041%
F/M ratio = 5.05

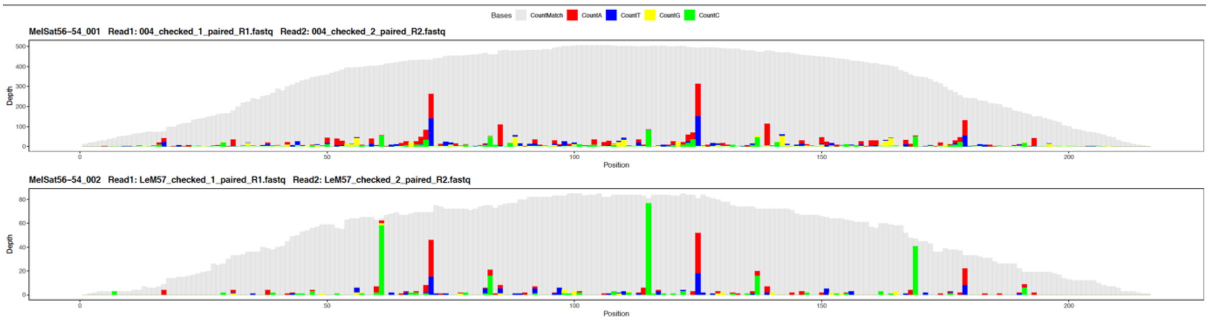

MelSat133-53
Divergence: F = 4.93% / M = 7.19%
Abundance: F = 0.0022% / M = 0.0005%
F/M ratio = 4.51

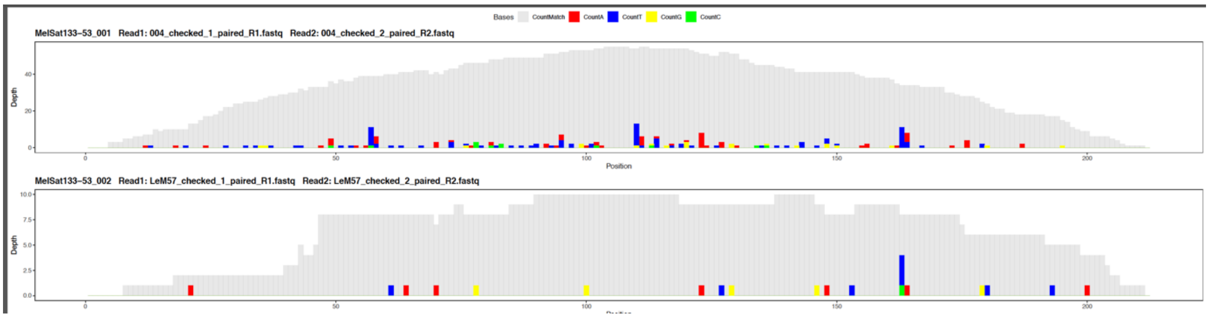

MelSat123-67
Divergence: F = 7.45% / M = 28.22%
Abundance: F = 0.0041% / M = 0.0012%
F/M ratio = 3.43

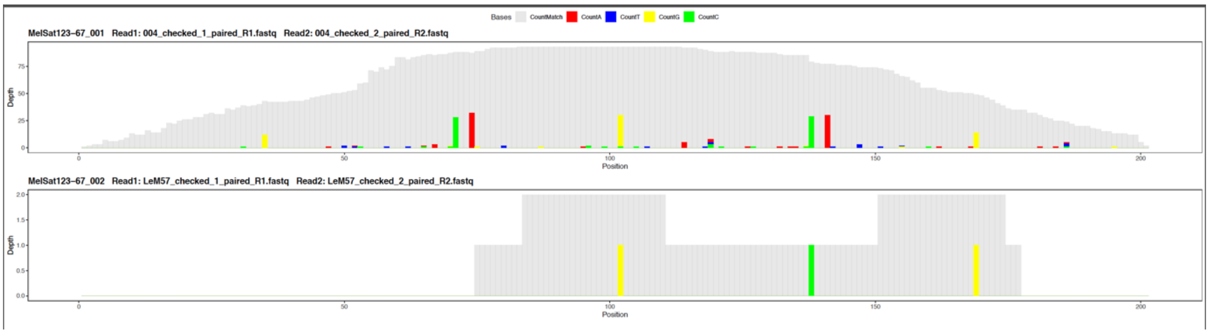

MelSat27-77
Divergence: F = 8.2% / M = 11.73%
Abundance: F = 0.0399% / M = 0.0123%
F/M ratio = 3.23

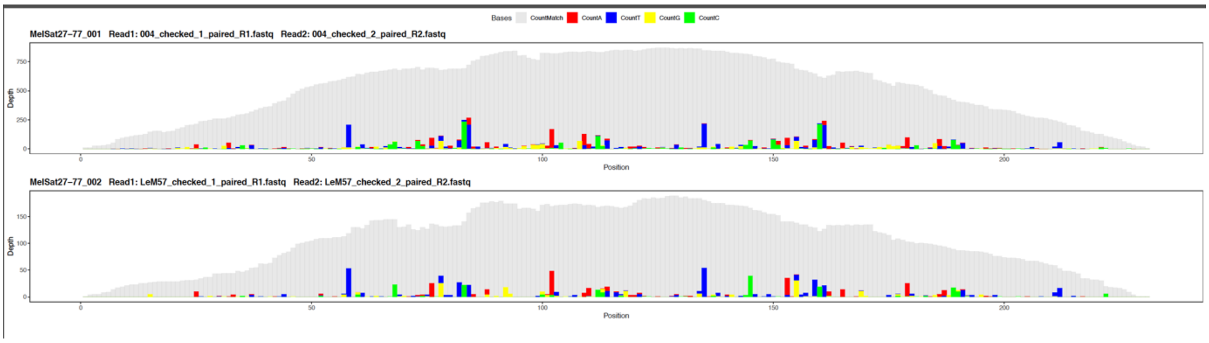

MelSat23-47
Divergence: F = 9.05% / M = 5.68%
Abundance: F = 0.0425% / M = 0.0158%
F/M ratio = 2.69

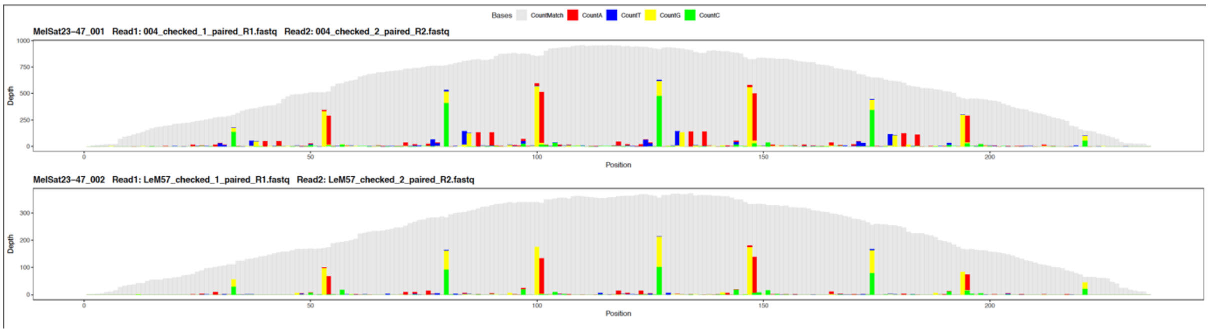

MelSat36-21
Divergence: F = 13.54% / M = 13.31%
Abundance: F = 0.0314% / M = 0.0121%
F/M ratio = 2.59

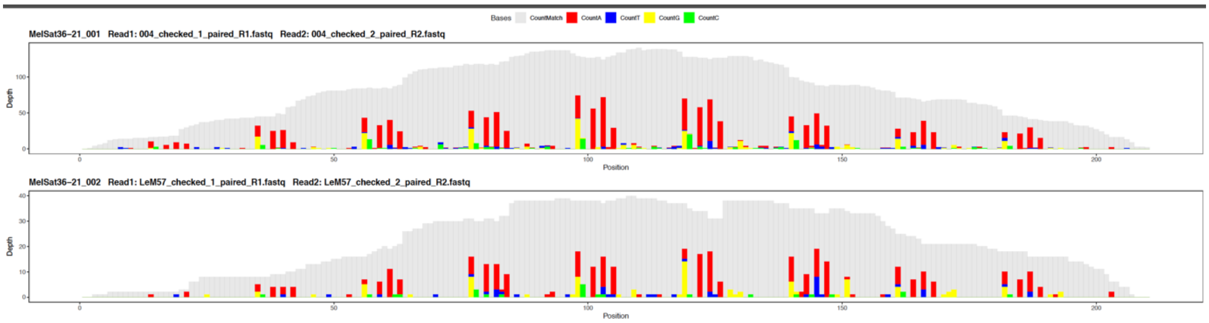

MelSat57-28
Divergence: F =12.61% /M = 25.38%
Abundance: F = 0.0203% / M = 0.0080%
F/M ratio = 2.53

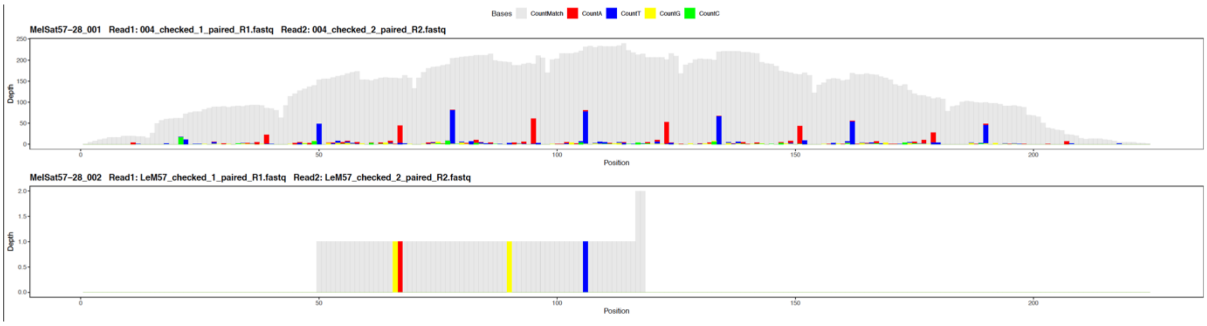

MelSat01-36
Divergence: F = 5%/M = 5.35%
Abundance: F = 0.4844% / M = 0.1991%
F/M ratio = 2.43

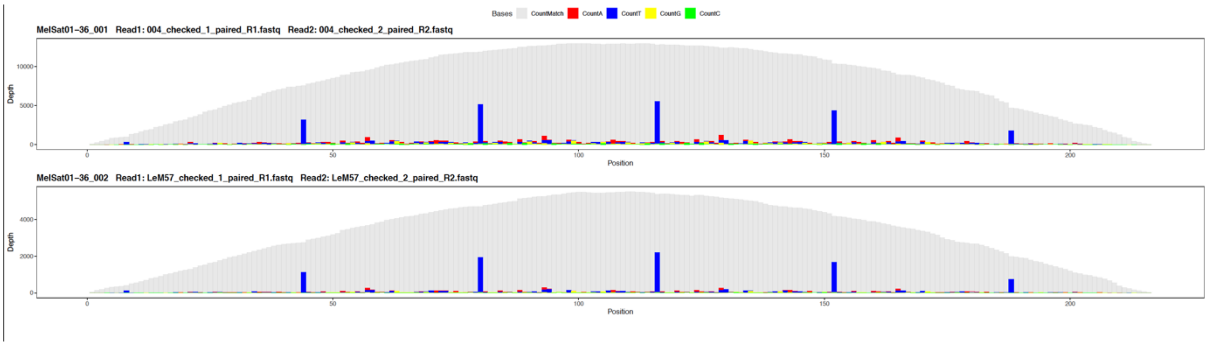

Supplement: Supplementary file 4 [file Image4.pdf]
