# Supplementary material for: Genomic Differences Between the Sexes in a Fish Species Seen Through Satellite DNAs
Source: Front Genet. 2021 Sep 30;12:728670. doi: 10.3389/fgene.2021.728670 (PMC8514694; doi:10.3389/fgene.2021.728670)

Supplementary Figure S2. Distribution of MelSat02-26 (A), MelSat22-34 (B), MelSat29-121 (C), MelSat44-52 (D) and MelSat52-38 (E) on male metaphases of *M. elongatus*. Scale bar = 10 µm.

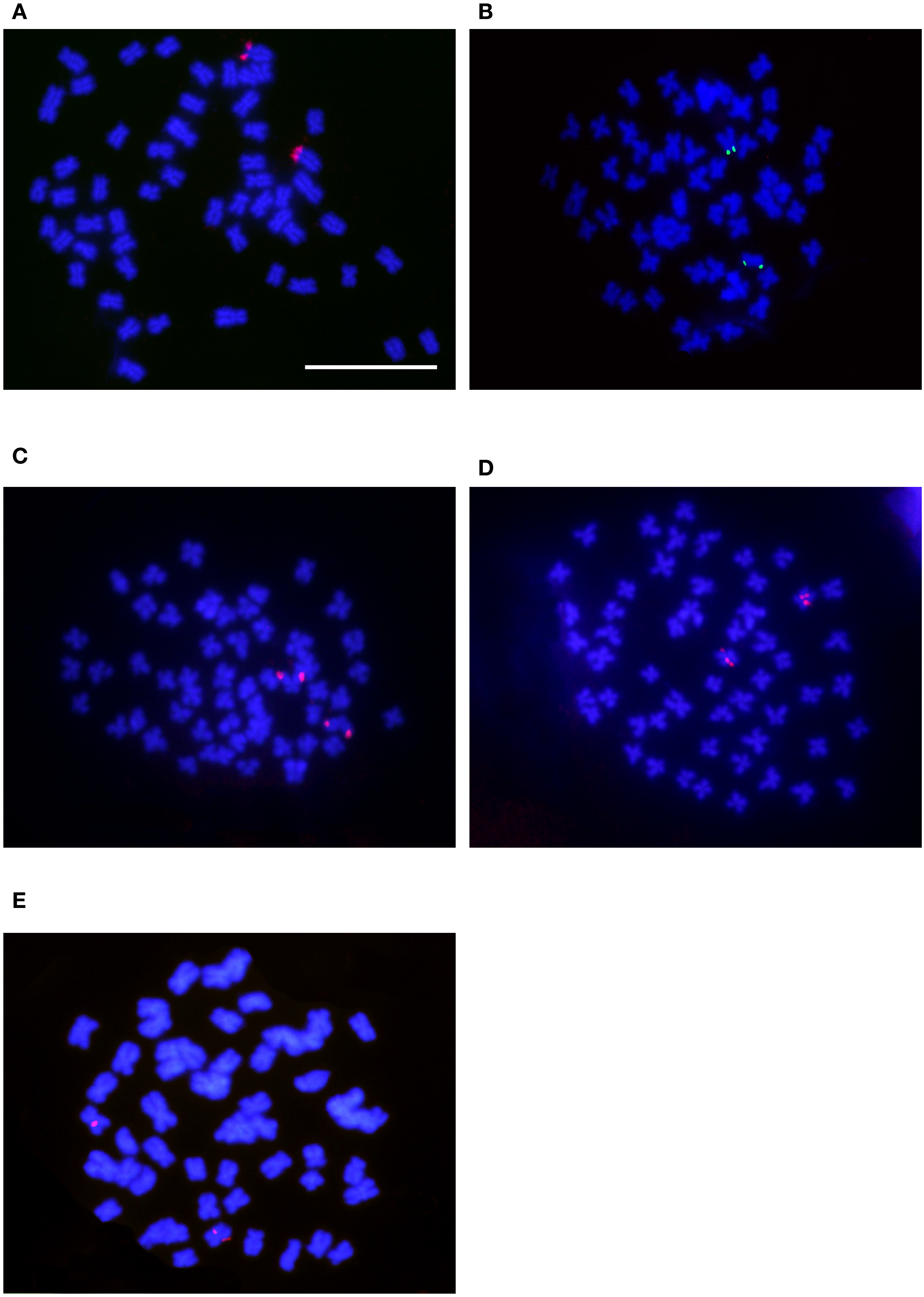

Supplement: Supplementary file 5 [file Image2.pdf]
