# Supplementary material for: Genomic Differences Between the Sexes in a Fish Species Seen Through Satellite DNAs
Source: Front Genet. 2021 Sep 30;12:728670. doi: 10.3389/fgene.2021.728670 (PMC8514694; doi:10.3389/fgene.2021.728670)

Supplementary Figure S3. Variant profiles for the 22 most male-biased satDNAs against their respective consensus sequences, arranged by most to least male-biased of the list according to the ratio quotient for F/M ratio. Topmost profiles correspond to the female copies and bottom profiles to the male ones for each satDNA. Divergence and abundance values for each selected satDNA are also specified. F = female; M = male.

MelSat124-53
Divergence: F = 7.11% / M = 6.27%
Abundance: F = 0.0041% / M = 0.0252%
F/M ratio = 0.163

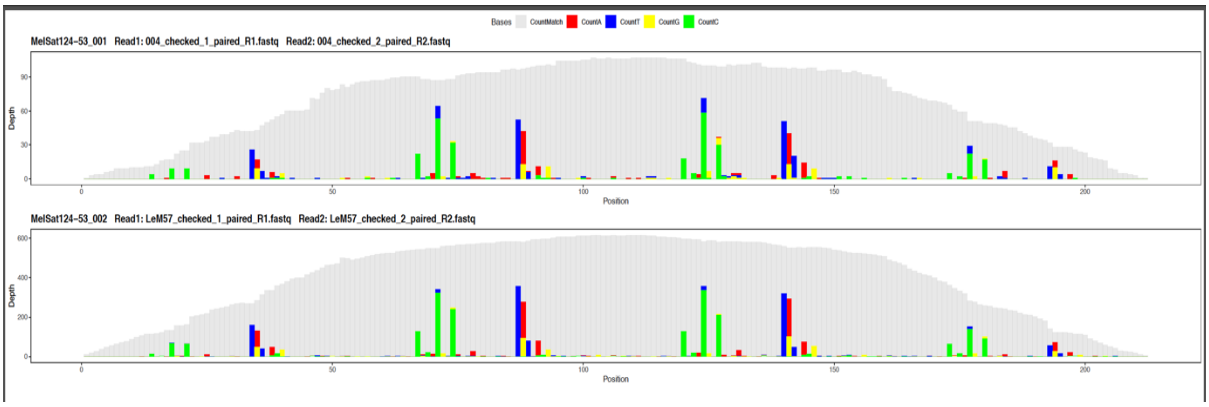

MelSat59-61
Divergence: F = 7.77% / M = 7.43%
Abundance: F = 0.0197% / M = 0.0962%
F/M ratio = 0.204

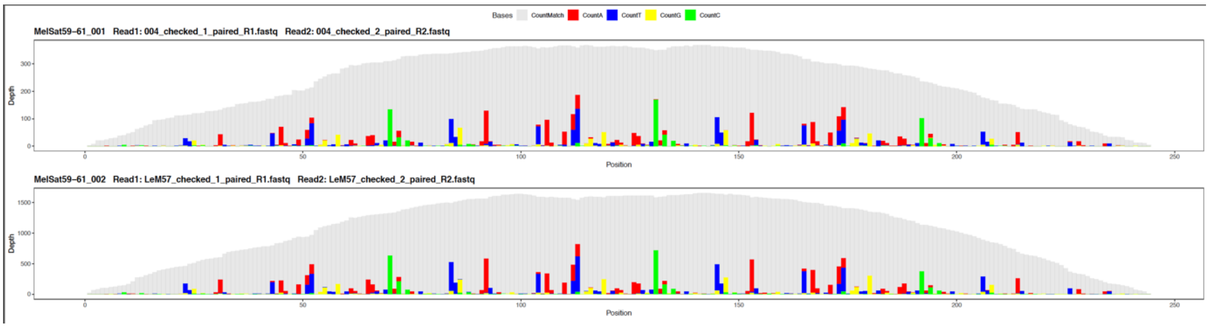

MelSat43-26
Divergence: F = 7.26% / M = 6.81%
Abundance: F = 0.0262% / M = 0.1059%
F/M ratio = 0.247

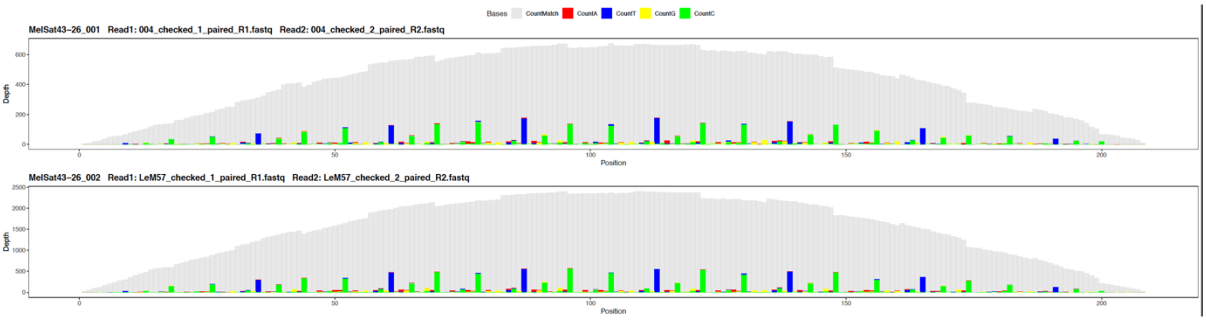

MelSat98-69
Divergence: F = 3.9% / M = 3.9%
Abundance: F = 0.0065% / M = 0.0169%
F/M ratio = 0.385

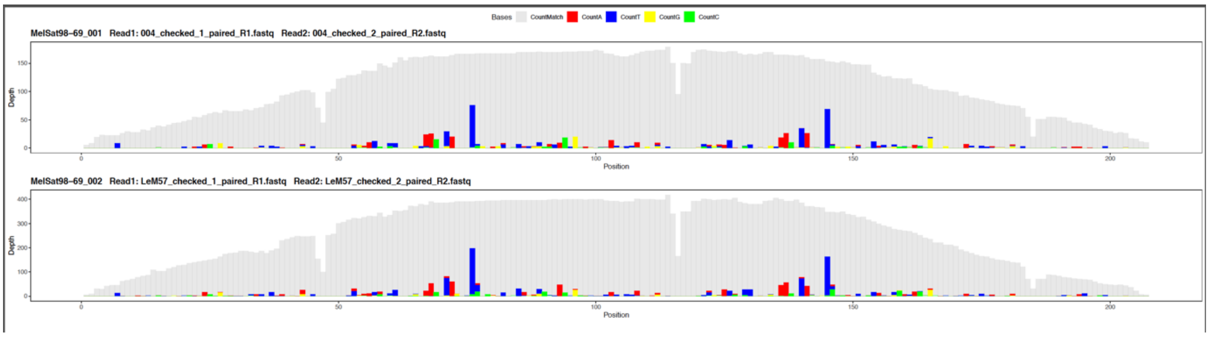

MelSat80-50
Divergence: F = 5.4% / M = 5.21%
Abundance: F = 0.0125% / M = 0.0307%
F/M ratio = 0.408

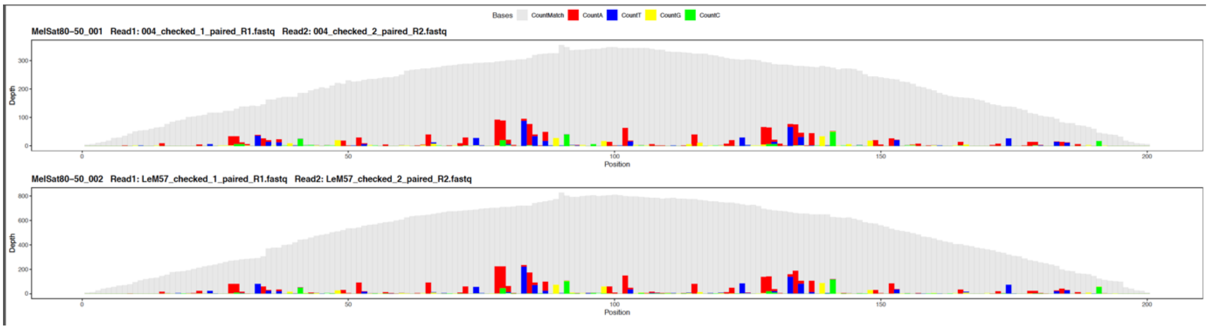

MelSat102-38
Divergence: F = 3.41% / M = 2.75%
Abundance: F = 0.0062% / M = 0.0150%
F/M ratio = 0.415

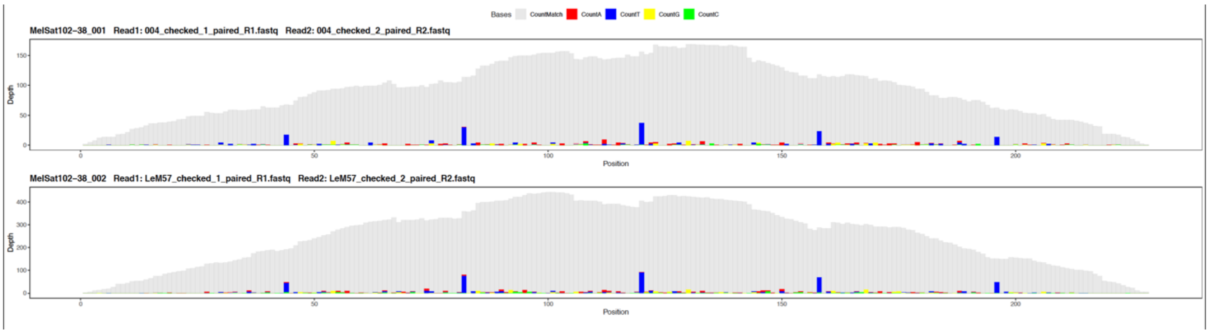

MelSat49-33
Divergence: F = 7% / M = 7%
Abundance: F = 0.0241% / M = 0.0571%
F/M ratio = 0.422

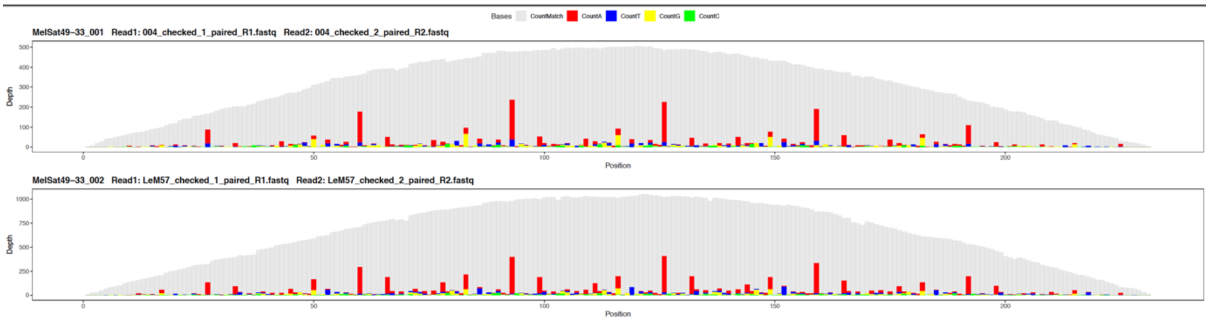

MelSat129-45
Divergence: F = 9.5% / M = 9.41%
Abundance: F = 0.0030% / M = 0.0064%
F/M ratio = 0.468

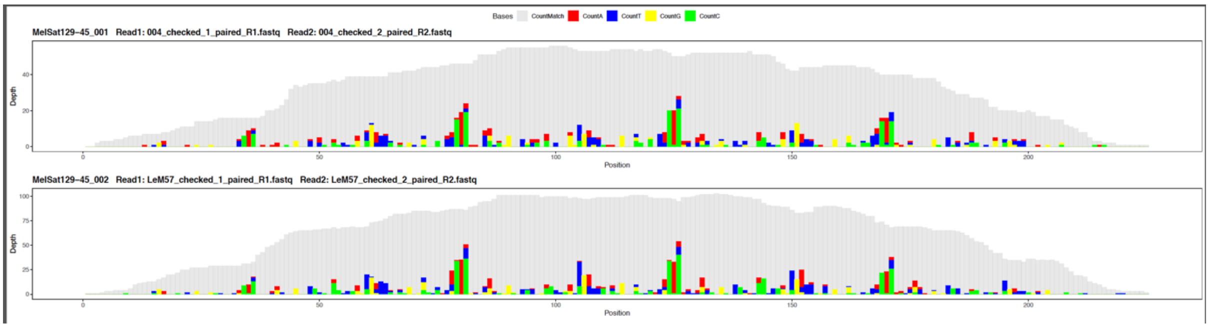

MelSat46-45
Divergence: F = F = 6% / M = 4.9%
Abundance: F = 0.0248% / M = 0.0516%
F/M ratio = 0.479

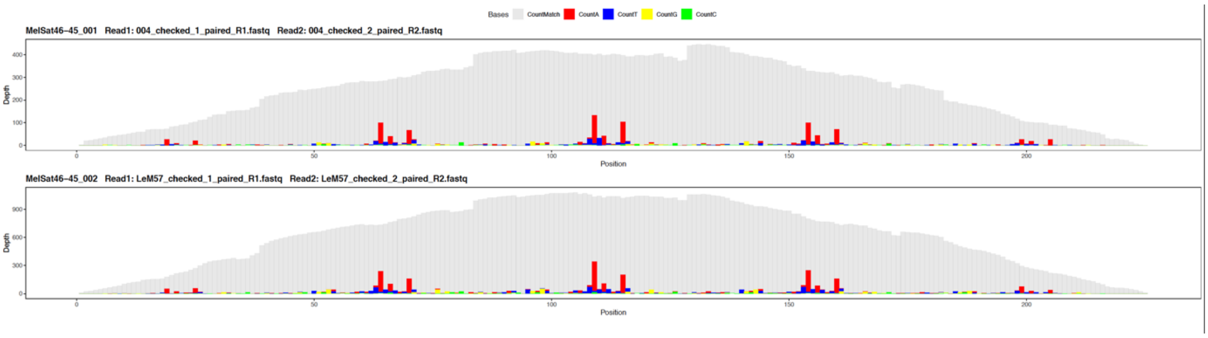

MelSat140-24
Divergence: F = 6% / M = 10%
Abundance: F = 0.0005% / M = 0.0010%
F/M ratio = 0.496

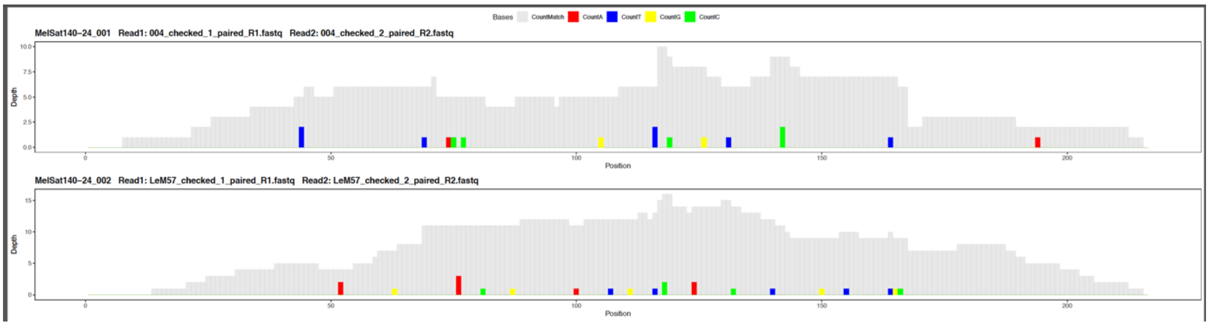

MelSat132-36
Divergence: F =5.49% / M = 4.74%
Abundance: F = 0.0023% / M = 0.0045%
F/M ratio = 0.503

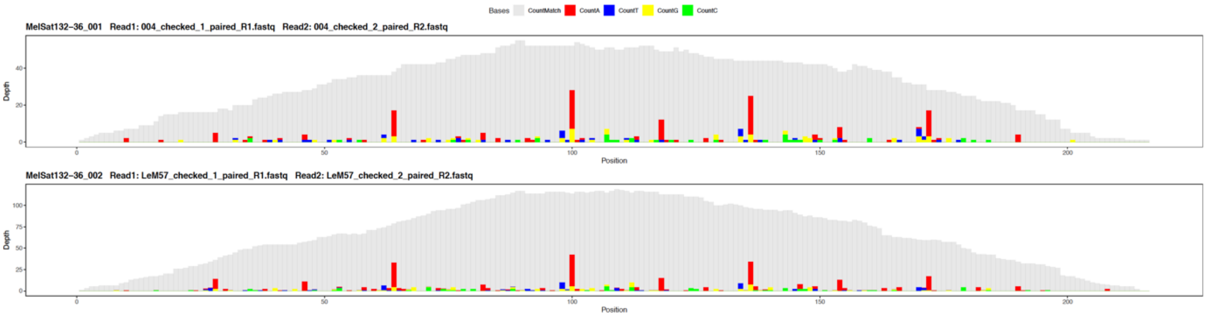

MelSat69-33
Divergence: F = 4.68% / M = 4.15%
Abundance: F = 0.0149% / M = 0.0287%
F/M ratio = 0.519

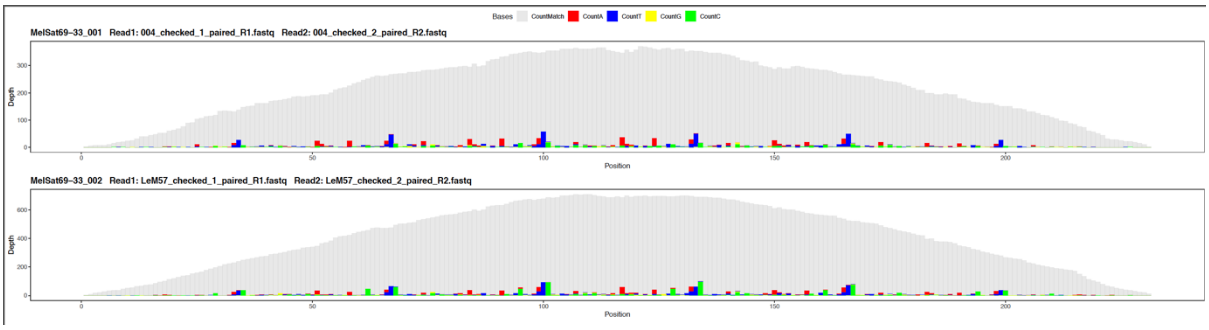

MelSat100-22
Divergence: F = 8.22% / M = 7.64%
Abundance: F = 0.0063% / M = 0.0116%
F/M ratio = 0.542

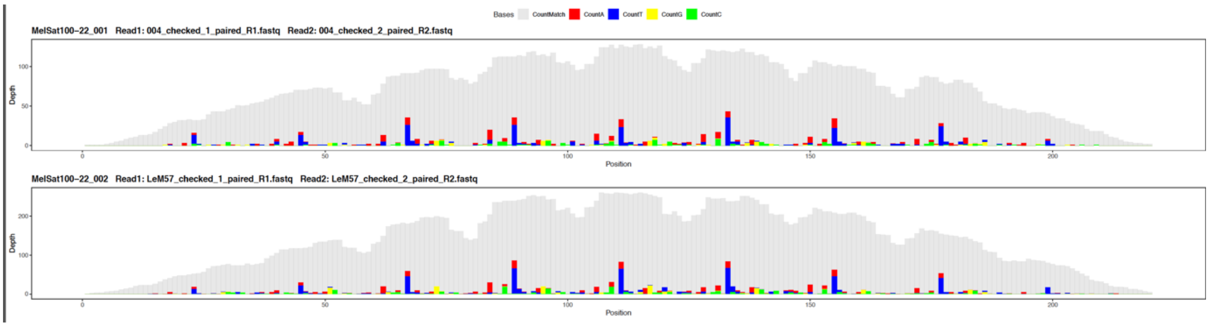

MelSat101-15
Divergence: F = 15.4% / M = 14.91%
Abundance: F = 0.0063% / M = 0.0114%
F/M ratio = 0.551

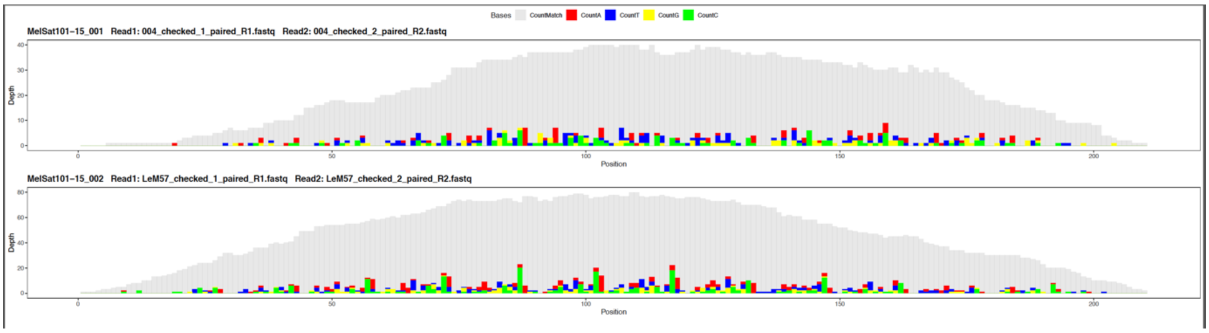

MelSat92-31
Divergence: F = 8.58%/ M = 7.76%
Abundance: F = 0.0082% / M = 0.0146%
F/M ratio = 0.561

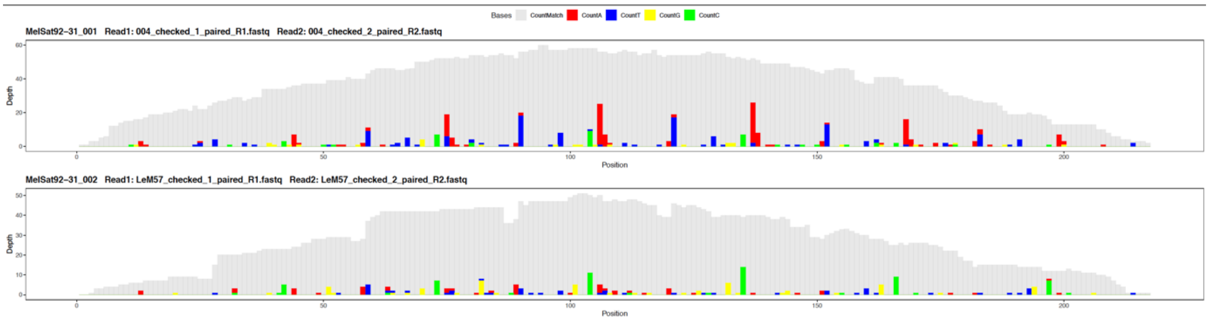

MelSat30-22
Divergence: F = 6.54% / M = 6%
Abundance: F = 0.0353% / M = 0.0626%
F/M ratio = 0.564

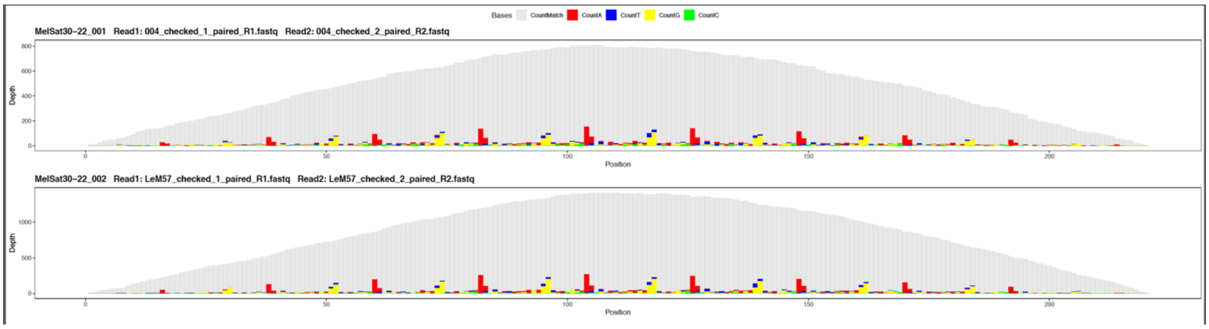

MelSat117-49
Divergence: F = 5.41% / M = 4.58%
Abundance: F = 0.0048% / M = 0.0085%
F/M ratio = 0.567

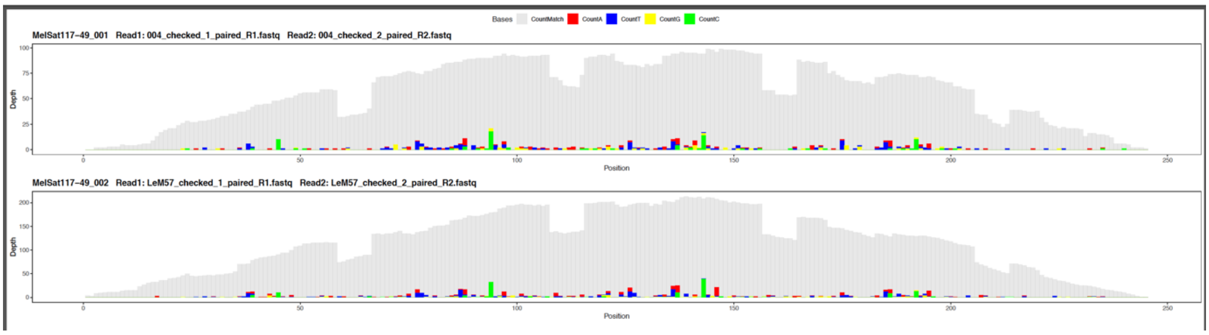

MelSat78-24
Divergence: F = 21.86% / M = 20.15%
Abundance: F = 0.0137% / M = 0.0237%
F/M ratio = 0.580

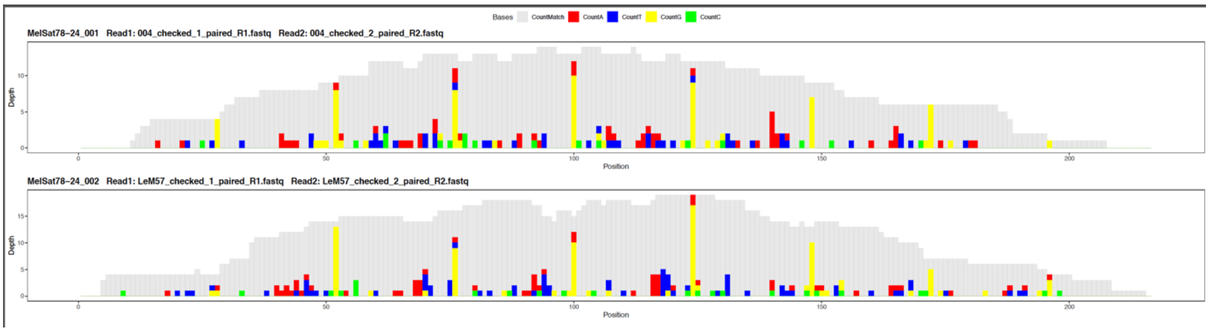

MelSat62-32
Divergence: F = 11.86% / M = 10.84%
Abundance: F = 0.0187% / M = 0.0321%
F/M ratio = 0.581

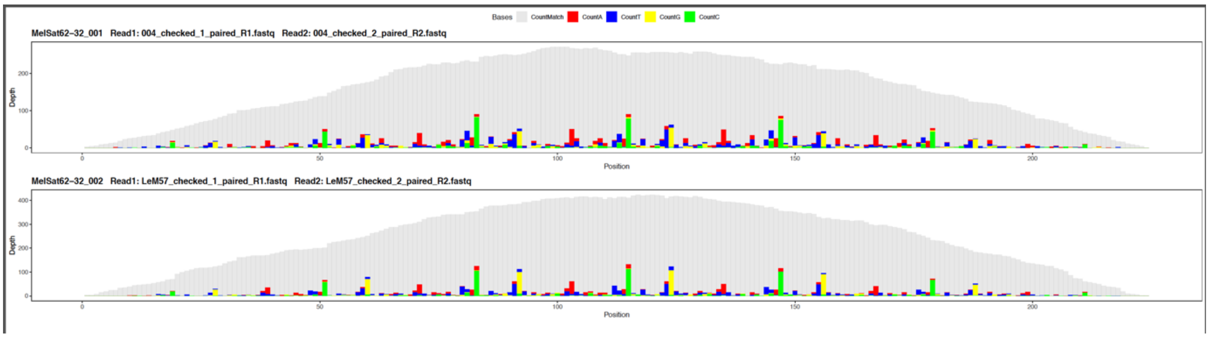

MelSat137-21
Divergence: F = 16.08% / M = 14.23%
Abundance: F = 0.0020% / M = 0.0034%
F/M ratio = 0.6034

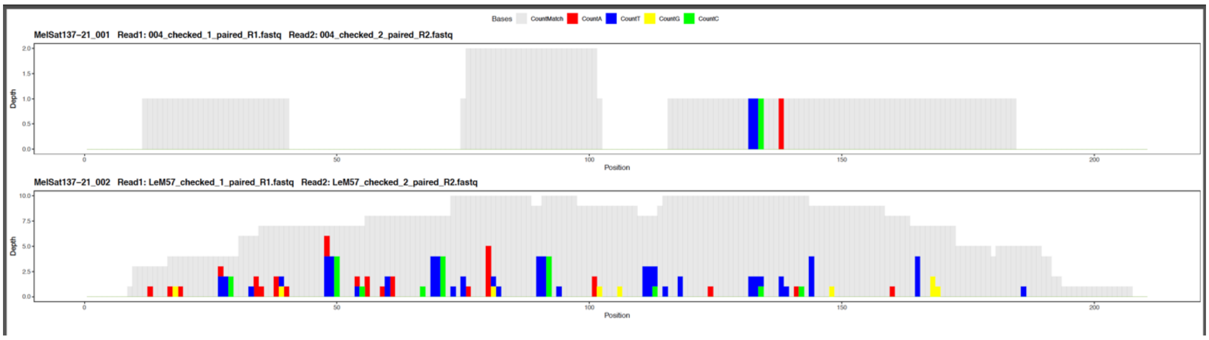

MelSat135-66
Divergence: F = 6.2% / M = 4.44%
Abundance: F = 0.0020% / M = 0.0034%
F/M ratio = 0.6039

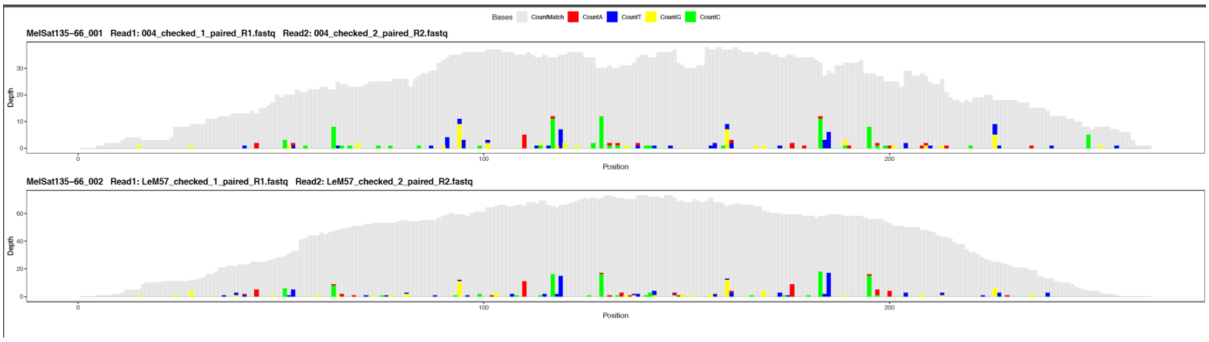

MelSat118-43
Divergence: F =25.82% / M = 16.21%
Abundance: F = 0.0046% / M = 0.0073%
F/M ratio = 0.633

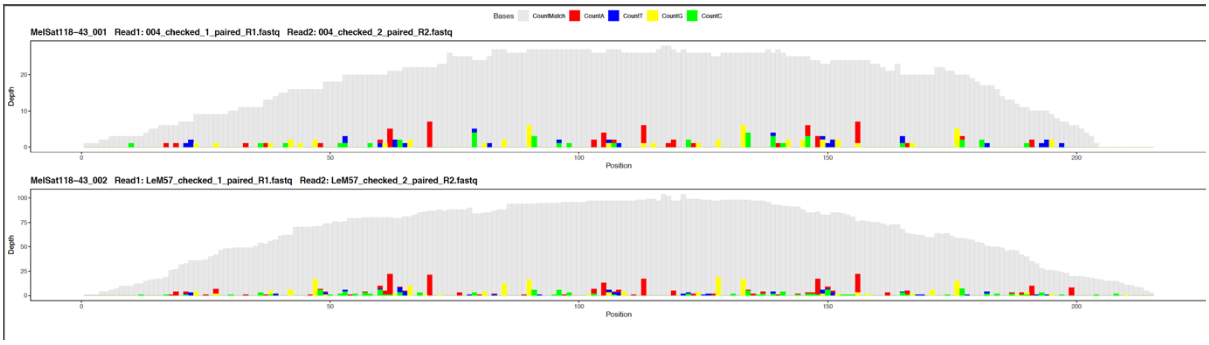

MelSat48-29
Divergence: F = 16% / M = 14%
Abundance: F = 0.0243% / M = 0.0382%
F/M ratio = 0.636

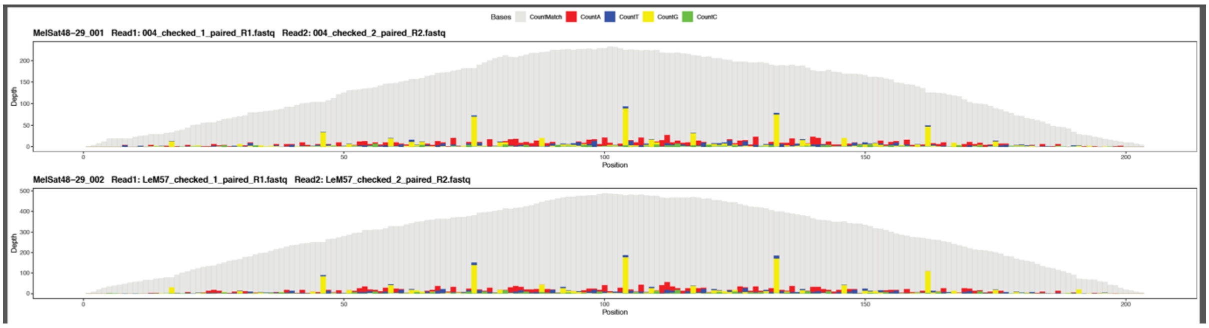

Supplement: Supplementary file 6 [file Image3.pdf]
