# Supplementary material for: Genomic Differences Between the Sexes in a Fish Species Seen Through Satellite DNAs
Source: Front Genet. 2021 Sep 30;12:728670. doi: 10.3389/fgene.2021.728670 (PMC8514694; doi:10.3389/fgene.2021.728670)

Supplementary Figure S1. Alignment between MelSat09-60 and MelSat113-60 characterising the only superfamily (SF1).

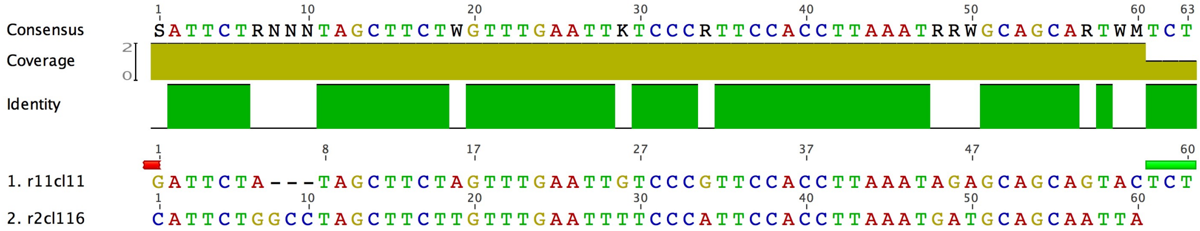

Supplement: Supplementary file 7 [file Image1.pdf]
